# Heat shock proteins: Biological functions, pathological roles, and therapeutic opportunities

**DOI:** 10.1002/mco2.161

**Published:** 2022-08-02

**Authors:** Chen Hu, Jing Yang, Ziping Qi, Hong Wu, Beilei Wang, Fengming Zou, Husheng Mei, Jing Liu, Wenchao Wang, Qingsong Liu

**Affiliations:** ^1^ Anhui Province Key Laboratory of Medical Physics and Technology Institute of Health and Medical Technology Hefei Institutes of Physical Science Chinese Academy of Sciences Hefei Anhui P. R. China; ^2^ Hefei Cancer Hospital Chinese Academy of Sciences Hefei Anhui P. R. China; ^3^ University of Science and Technology of China Hefei Anhui P. R. China; ^4^ Precision Medicine Research Laboratory of Anhui Province Hefei Anhui P. R. China

**Keywords:** cancers, heat shock proteins, molecular chaperone, proteostasis, target therapy

## Abstract

The heat shock proteins (HSPs) are ubiquitous and conserved protein families in both prokaryotic and eukaryotic organisms, and they maintain cellular proteostasis and protect cells from stresses. HSP protein families are classified based on their molecular weights, mainly including large HSPs, HSP90, HSP70, HSP60, HSP40, and small HSPs. They function as molecular chaperons in cells and work as an integrated network, participating in the folding of newly synthesized polypeptides, refolding metastable proteins, protein complex assembly, dissociating protein aggregate dissociation, and the degradation of misfolded proteins. In addition to their chaperone functions, they also play important roles in cell signaling transduction, cell cycle, and apoptosis regulation. Therefore, malfunction of HSPs is related with many diseases, including cancers, neurodegeneration, and other diseases. In this review, we describe the current understandings about the molecular mechanisms of the major HSP families including HSP90/HSP70/HSP60/HSP110 and small HSPs, how the HSPs keep the protein proteostasis and response to stresses, and we also discuss their roles in diseases and the recent exploration of HSP related therapy and diagnosis to modulate diseases. These research advances offer new prospects of HSPs as potential targets for therapeutic intervention.

## Graphical abstract

Heat shock proteins (HSPs) are in charge of proteostasis and buffer the stresses. Malfunction of HSPs lead to many diseases. In this review, we discuss the current understanding about the functions and structures of the major HSP families, the HSP network in proteostasis and their roles in diseases, and the prospects of these proteins as potential targets for therapeutic intervention.

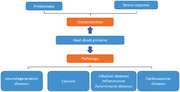

## 1 INTRODUCTION

In living organisms, cells are under constantly changing conditions and maintenance of cellular protein homeostasis is crucial for cell survival and integrity,^1^, ^2^ as protein misfolding and aggregation have been found to induce malfunction of proteins and lead to various diseases.^3^, ^4^ The ubiquitous and conserved heat shock proteins (HSPs), which exist in both prokaryote and eukaryote organisms, belong to a large family of proteins that are in charge of proteostasis.^5^ They function in a wide range of cellular housekeeping processes, including the folding of newly synthesized polypeptide, refolding metastable proteins, protein complex assembly, the degradation of misfolded proteins, and dissociating protein aggregates.^5^, ^6^ Under normal conditions, HSPs account for 5–10% of the total cellular protein content and they work as an integrated network to maintain proteostasis.^5^ Although under extreme conditions, the heat shock transcription factors (HSFs) are activated in response to stresses, leading the transcription of abundant HSPs to buffer the stresses.^7^, ^8^ And based on the primary molecular chaperon function, they also participate in multiple processes in eukaryotic cells, and malfunction of HSPs has reported to related with many diseases. In this review, we discuss the current understanding about the structures features and the chaperon functions of the major HSPs, and the regulation of HSPs by their cochaperons, describe the HSP network in proteostasis and how they response to the stresses. We also discuss their roles in cancers, neurodegeneration, autoimmune, inflammation, infection and cardiovascular diseases (CVDs), and summarize the prospects of these proteins as potential targets and biomarkers for therapeutic intervention, the development, and shortcomings of HSP inhibitors.

## 2 THE DISCOVERY OF HSPs

The protective ability of HSPs to cope with stress was discovered long before the function was understood. The chromosomal puffing induced by temperature shock was first reported on fruit fly in 1962,^9^ and in the following years, similar chromosomal puffs were observed in different organisms including both prokaryotes and eukaryotes, and the puffing happened not only after heat shock, but also in response to other survival pressures, such as hypoxia, ischemia, reactive oxygen species (ROS), or endotoxins.^10^ It indicates that this phenomenon executes protective function for cell survival under diverse stresses.^10^, ^11^ Moreover, the newly transcribed messenger RNA at the puff site induces the synthesis of HSPs in vivo.^12^, ^13^ Since then, identification and functional research of HSPs became the focus in this area.

Among the many HSPs, HSP60/70/90 families are the most studied HSPs. GroEL, the bacterial homolog of HSP60, was first reported in 1973 and found to participate in bacteriophage assembly.^14^, ^15^ Later, HSP60 homologs in plant chloroplast and in yeast mitochondria were also identified, and they facilitate the folding of peptides with adenosine triphosphate (ATP) and cofactor chaperonin‐10, and mediate protein importation into yeast mitochondria, indicating a role of HSP60 in protein folding and assembly.^16^, ^17^ In the few years later, the mechanism of GroEL‐GroES (as homologues of HSP60‐HSP10 in prokaryotes) folding machine was identified as a heptameric rings that encapsulate the substrates inside the complex and perform protein folding by ATP hydrolysis.^18^, ^19^ For HSP70 family, the first heat shock protein HSP70 was discovered in drosophila, then the *Escherichia coli* homolog of HSP70‐DnaK was found in the 1980s. In the later 1980s, evidence emerged that HSP70 can interact with the hydrophobic peptides and associate with nascent polypeptide in translation,^20^, ^21^ which was followed by the discovery of another two factors regulating the ATPase activity of HSP70, DnaJ and GrpE.^22^ By reconstitution, the protein folding pathway in vitro, it was then found that protein folding is a highly organized cellular process that needs the cooperation of HSP70 and HSP60‐HSP10.^23^ At this time, the molecular chaperon function of HSPs has been defined as to maintain the correct folding of polypeptide chains and assembly of protein complex.^21^, ^24^ In 1983, the first HSP90 gene was isolated from yeast, and then HSP90 protein was reported to associate with steroid receptors and Src kinase. This association is required for the functions of these proteins, as reduced HSP90 compromise the activity of steroid receptors.^25^, ^26^ Then in 1990s, it was revealed that HSP90 functions as molecular chaperones and participate in the folding of client proteins.

Later, it was observed that the HSPs are high evolutionarily conserved from bacteria to eukaryote species.^27^, ^28^, ^29^, ^30^ Since then, increasing understanding about the mechanism of HSPs were obtained by structural biology analysis, and the functions of HSPs expanded to almost every aspect in the life of proteins from de novo synthesis to degradation.^31^, ^32^

## 3 HSPs CLASSIFICATION

Based on molecular weights, HSPs are classified into large HSPs, HSP90, HSP70, HSP60, HSP40, and small HSPs families, the functions of which are involved in the entire metabolic process of proteins (Table 1). They function as holdase, foldase, sequestrase, aggregase, or disaggregase, and work as a network to keep the proteostasis in cells and also an efficient first line of defense in response to stress.^5^, ^33^

**TABLE 1:** Brief summary of heat shock protein families’ members, cellular locations, and functions

Family	Major Members and location		Characteristics	function	Reference
Small HSPs	HSPB1‐HSPB10	Cytosol Mitochondria Nucleus	ATP‐independent large heterogeneous oligomers	Work as holdase to prevent aggregation; sequestrate misfolded proteins;	^72^, ^80^, ^81^
HSP40/DNAJ	DNAJA DNAJB DNAJC	Cytosol mitochondria nucleus	J domain containing proteins Interact with HSP70	Serve as cochaperon of HSP70, regulating HSP70 ATPase activity; Hold misfolding substrates and recruit HSP70;	^22^, ^40^, ^43^
HSP60	HSP60	Mitochondria	Double ring structure contains Two heptameric rings of HSP60, cooperates with HSP10; ATP‐dependent	Protein foldase Prevents aggregation	^18^, ^62^, ^82^, ^83^
	TRiC	Cytosol	Double ring structure contains two octameric rings of TRiC; ATP‐dependent.		
HSP70	HSPA1A/1B HSPA1L HSPA2 HSPA6 HSPA7 HSPA8 HSPA12A/12B HSPA13 HSPA14	Cytosol Nucleus	Conserved structure containing NTD‐SBD domains; Chaperon function based on the allosteric conformation change cycle in ATP‐dependent way	Multiple functions in proteostasis Work as holdase, foldase, prevent aggregation and triage the protein fates	^34^, ^35^, ^84^
	HSPA5	ER			
	HSPA9	Mitochondria			
HSP90	HSP90AA HSP90AB GRP9 TRAP1	Cytosol Cytosol Cytosol/ER Mitochondria	Form homodimer and undergoes allosteric open‐closed conformation change in the folding process; ATP dependent	Foldase for proteins de novo synthesized and refold misfolded proteins; major substrates include kinases, steroid receptors	^4^, ^54^, ^85^
Large HSPs	HSP110 GRP170	Cytosol ER	Belong to HSP70 superfamily	Holdase; keep proteins from aggregation; Cochaperon of HSP70	^47^, ^48^, ^77^

### 3.1 HSP70

HSP70 /DnaK are conserved molecular chaperon proteins ubiquitously expressed in prokaryote and eukaryote organisms.^34^ DnaK is expressed in the prokaryotes, and HSP70 is the eukaryote counterpart. In human genome, there are about 17 genes encoding multiple HSP70 proteins.^35^ The expression of some of them is stress induced, called HSP70s, which activate to deal with the environment changes, the others are housekeeping HSC70 (heat shock cognate) that are constitutively expressed.^34^, ^35^, ^36^ HSP70s are expressed in different cellular locations, including cytosol, nucleus, endoplasmic reticulum (ER), mitochondria, and secreted, and they keep the dynamic balance of the synthesis, folding, degradation, and translocation of proteins.^34^, ^35^, ^36^

**FIGURE 1:**
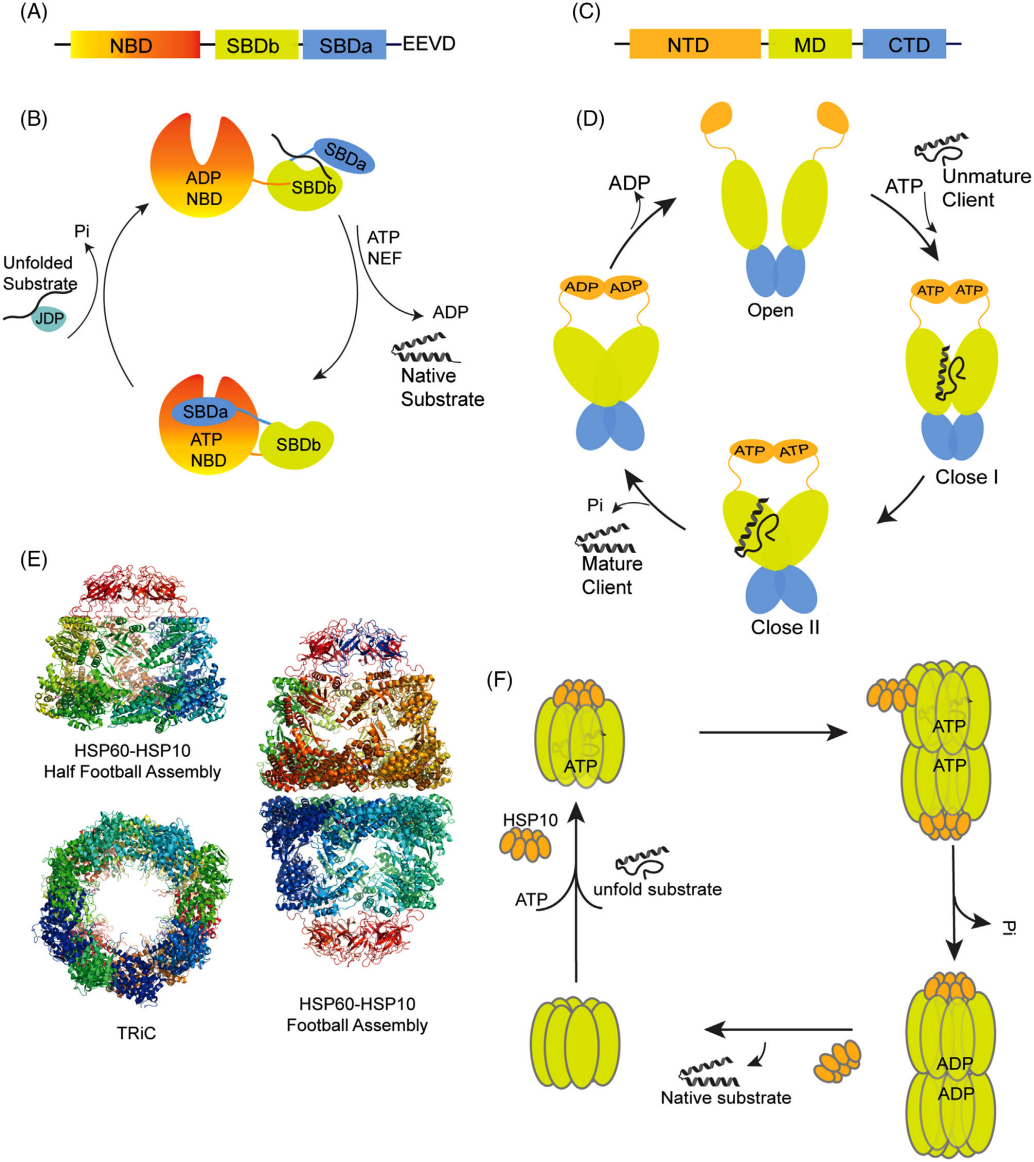
(A) Schematic diagram of the HSP70 domains. (B) The conformational cycles of HSP70. (C) Schematic diagram of the HSP90 domains. (D) The ATP‐dependent chaperone cycles of HSP90. (E) The structures of half football (PDB ID: 6MRD)^63^ and full football assembly (PDB ID: 6MRC)^63^ of HSP60‐HSP10, TRiC (PDB ID: 4A0O),^64^ images in Figure 1E adapted from Refs. 63 and 64. (F) The model of HSP60‐HSP10 chaperone cycles

HSP70 proteins shares conserved structure features. The N‐terminal nucleotide binding domain (NTD) ligate to the C‐terminal substrate binding domain (SBD) by a flexible linker. SBD domain includes two subdomains, SBDα and SBDβ, followed by a C‐terminal tail with EEVD motif (conserved Glu‐Glu‐Val‐Asp, EEVD) that recognizes several cochaperones (Figure 1A).^34^ The chaperon activity of HSP70 is based on the allosteric conformation change of HSP70, which is ATP‐dependent. In this process, ADP‐bound HSP70 recognizes the substrate at the SBD domain with high affinity. For DnaK in *E. coli*, it prefers peptides with a hydrophobic core of five residues,^37^ and for HSP70, it seems to be more promiscuous for the substrate recognition.^38^ Then nuclear exchange factors (NEFs) mediate the exchange from ADP to ATP, and ATP induces the rotation of HSP70 at the NBD lobes and the SBDα helix lid covers on the NBD. This ATP‐bound conformation of HSP70 has low affinity for substrates, causing the release of substrates.^34^, ^39^ Upon the interaction with the cochaperone J‐domain protein (JDP/HSP40) with HSP70, HSP70 undergoes ATP hydrolysis and returns to the ADP‐bound mode, triggering another round of cycle.^40^, ^41^ The released substrate could rebind HSP70 and undergoes further folding cycles until it reaches its native state (Figure 1B).

The functions of HSP70 are regulated by the cochaperones binding to HSP70 at different domains.^42^ The major cochaperones coupling with HSP70 NBD are J domain protein (JDP)/HSP40 and nucleotide exchange factor (NEF) families. The JDP/HSP40 families are not only chaperones by themselves, but also act as cochaperone of HSP70.^43^ JDP/HSP40 recognize the hydrophobic features on the unfolded or nascent proteins and hand over the substrates to HSP70.^43^ In addition, HSP70‐HSP40 complex elevates the HSP70 ATPase and folding activity, facilitating the binding with substrates, and preventing aggregates.^40^ Another family of ubiquitous cochaperones that bind to HSP70 are NEFs, which in eukaryotes include three major classes, HSP110, BAG, and HSPBP1. NEFs interact with HSP70 at the NBD domain and mediate the dissociation of ADP and the rebinding of ATP. Different NEFs coupled with HSP70 serve diverse cellular functions. In the BAG family, BAG1 has been reported to mediate HSP70 with the proteasomal degradation pathway, whereas BAG3‐HSP70 complex is a hub for controlling protein aggregation.^44^, ^45^, ^46^ HSP110 not only functions as a chaperon protein, but also is the most abundant NEFs of HSP70, promoting protein disaggregation.^47^, ^48^ Besides HIP protein that not belongs to the JDP and NEFs family also interacts with HSP70 at the NBD domain. HIP competes with BAG1 for HSP70 binding, and prevents the nucleotide exchange, which delays the release of substrate, indicating that it is important to avoid the premature substrate release.^42^ Meanwhile, the C‐terminal EEVD motif of HSP70 interacts with E3 ligase CHIP, mediating the substrate degradation through ubiquitin‐proteasome system (UPS).^49^

### 3.2 HSP90

HSP90s are highly conserved HSPs with molecular weight of 90 kDa. They are ubiquitously expressed in all species except for archaea. In human genome, there are six genes encoding HSP90, which are either expressed in cytosol (induced expressed HSP90AA and constitutive expressed HSP90AB),^50^, ^51^ ER (GRP94), or mitochondrial (TRAP) (Table 1).^52^, ^53^, ^54^

All the HSP90 homologues share common structure elements. The HSP90 monomer contains three domains, including nucleotide‐binding domain (NTD) at N‐terminal, a middle domain (MD), and a C‐terminal domain (CTD) driving the homodimer of two subunits of HSP90 and contains an Met‐Glu‐Glu‐Val‐Asp (MEEVD) motif for cochaperone coupling (Figure 1C).^55^ HSP90 folding activity is based on ATP‐dependent dynamic conformation changes and regulated by the multiple cochaperones. Without nucleotide bound, HSP90 stays in an NTD‐open conformation, forming dimers through the CTD domain. The unfolded client proteins are loaded to HSP90 in open conformation at the MD domain, and ATP binding induces the dimerization of NTD. The following ATP hydrolysis leads to a more twisted and closed conformation of HSP90, and ADP is released from HSP90, turning the conformation back to open mode. By repeating this cycle, the client proteins are folded and released in native conformations (Figure 1D).^56^ In human proteome, about 10% proteins are HSP90 clients and their maturation is dependent on HSP90. By analyzing the clients’ categories by mass spectrum, Barrios‐Rodiles et al. found that HSP90 interacts with the relatively abundant proteins in human proteome, including transcription factors, ubiquitin proteins, and about 60% kinase proteins in human kinome.^57^

In eukaryotes, many cochaperones of HSP90 have been found to regulate the function of HSP90. Some cochaperones adjust the ATPase activity of HSP90. For example, AHA1 is a cochaperone that stimulates the ATPase activity; meanwhile, p23 suppresses HSP90 ATPase activity by allosterically switching the conformation of the catalytic loop in HSP90.^58^ Several tetratricopeptide repeat (TPR) domain‐containing cochaperones interact with the MEEVD peptide of HSP90, the most studied TPR protein is HSC70/HSP90‐organizing protein HOP, which stabilizes HSP90 in open conformation and behaves as a linker for substrate handover from HSP70 to HSP90.^59^ The recognition for substrates also depends on general cochaperons, such as cochaperone CDC37, which is a universal cochaperone for kinase‐HSP90 interaction. Cochaperones also take part in the protein triage, as CHIP and Sgt1 are required for efficient proteasomal degradation of misfolded proteins.

HSP90 proteins function not only as protein foldases, but also participate the protein conformational maturation. For example, the structure stabilization of oncogenic kinases with activation mutations depends on HSP90 chaperon. Exposure to HSP90 inhibitor leads to the instability and degradation of kinase clients through proteasome. Due to its extensive involvement of the protein stability and activity, HSP90 plays many roles in cell survival derived from the chaperone function, such as cell signaling and cell cycle regulation.

### 3.3 HSP60

HSP60 is one of the most conserved proteins expressed in all species, and is also named as chaperonin or Cpn60. Two groups of chaperonins are found to be expressed in different locations in cells. Group I HSP60s are expressed in prokaryotes (known as GroEL) and in the eukaryotic mitochondria and chloroplasts, and they work with cofactor HSP10 (GroES in prokaryote). Group II HSP60s are distributed in archaea and the eukaryotic cytosol (TRiC or CCT).^60^

The two types of chaperonins adopt similar conformation and substrate folding mechanism, both of which assist the protein folding through the ATP‐dependent cylinder folding cage. The group I GroEL‐GroES system is composed of three domains, an equatorial domain including ATP binding pocket, an apical domain, and an intermediate hinge domain in between. GroEL assembles into a back‐to‐back double ring structure with seven subunits in each ring.^61^ Compared to the GroEL‐GroES complex, the eukaryotic HSP60‐HSP10 system shows some different features. The double ring structure of HSP60 in eukaryotes depends on the cofactor and nucleotide interaction. HSP60 exists as a single heptamer ring without HSP10 and ATP. In an ATP‐dependent manner, the double‐ring structures are formed between single rings and HSP60/HSP10 forms football‐type complexes (Figure 1E).^62^, ^63^ For group II chaperonins, TRiC works independent of cochaperone HSP10(Figure 1E). An extra domain in the apical domain of TRiC forms an iris‐like structure that functions as the lid of TRiC. TRiC forms octamer complex and utilizes a mechanism for protein folding similar to that of HSP60‐HSP10.^64^ Upon ATP binding, heptamer HSP60 complex with HSP10 on the head region and the substrates are captured into the lumen of GroEL, after which the substrate is folded in the nanocage of HSP60 through ATP hydrolysis. Afterward, ATP binding with GroEL on the other ring induces the release of ADP and GroES, which allows the release of folded substrate (Figure 1F). Meanwhile, inadequately folded proteins can be encapsulated into GroEL again for further folding.^61^, ^65^ Chaperonin plays multiple roles in cells. For example, TRiC participates in the folding of about 10% new synthesized proteins.^66^ Chaperonin also prevents the aggregation and refolding the denatured protein under stress.^67^ Mitochondrial HSP60 is essential for maintaining the mitochondrial respiratory‐chain integrity. Meanwhile, HSP60s are dual‐directional regulators of apoptosis in response to extracellular or intracellular stresses.^68^, ^69^, ^70^

### 3.4 Small HSPs

sHSP/HSPB are ATP‐independent molecular chaperones defined by their low molecular weights (12–43 Kda). Until now, about 10 small HSPs have been found in the cytosol and nucleus of mammalian cells with diverse functions as holdase, sequestrase, or aggregase.^71^ Small HSPs are constitutively expressed to avoid protein aggregation, and they are upregulated rapidly under stress to buffer the protein unfolding pressure.^72^

The common structural feature of small HSPs is the conserved α‐crystalline domain,^73^ which is a β‐sandwich structure composed of seven or eight antiparallel β sheets.^72^ On the N‐ and C‐side of ACD is the N‐ and C‐terminal regions (NTEs and CTEs), which are variable in sequence and length, and contribute to the flexibility of the small HSP structures.^71^ In vivo, the HSPs form transient and plastic large oligomers as hollow spheres. During the formation of oligomers, sHSP monomers interact with each other through the ACD to form sHSP dimers.^74^ Then using sHSP dimer as basic construction material, the oligomers assemble in diverse modes. In human HSPB2/B3, the NTE recognizes the groove formed in the ACD dimer.^75^ While in other HSPs, such as *Triticum aestivum* HSP16.9, the dimers form higher oligomers through the NTE interactions between dimers.^76^ In brief, the oligomers are dynamic and exchangeable depending on the stress or temperature. Unlike the foldase activity of HSP90/70/60 chaperone, sHSPs have a distinct holdase activity that is ATP‐independent. sHSPs dimers interact with the early‐unfolded intermediate substrates and sequestrate them in the core of the sHSP/substrate complexes to avoid aggregation. Because sHSPs do not have foldase activity, the sequestrated substrates can be triaged for refolding by HSP70/90 chaperon system. Meanwhile, some other sHSPs have aggregase activity and promote ubiquitin‐dependent aggregation of substrates under diverse stresses.

### 3.5 Large HSPs

The large HSPs, including HSP110 and Grp170, are HSPs with large molecular weights found in eukaryotes. HSP110 is universally distributed in both cytosol and nucleus, whereas Grp170 is specially located at ER. They are evolutionarily conserved and homologous to the HSP70 family, thus often categorized as members of the so‐called “HSP70 superfamily.” Both of them are expressed constitutively in cells, and under stresses, they can also be induced rapidly to buffer the proteostasis.^77^ They work as holdases to prevent the aggregation of substrates. The yeast HSP110 (sse1p) also mediates both the ubiquitin‐dependent and ubiquitin‐independent degradation by interacting with the 19S subunit of the 29S proteasome.^78^ Meanwhile, HSP110 is a cochaperon of HSP70, acting as NEF for the ADP‐ATP exchange. Cochaperone HSP‐110 is not only important for the cellular protein folding capability of HSP70, but also essential for the HSP70 disaggregation machinery and participates in the HSP70‐mediated degradation pathway.^79^

## 4 DIVERGENT FUNCTIONS OF HSPSs

HSPs are widely distributed in cells and participate in a variety of cellular processes to keep cell integrity, maintain protein homeostasis, and response to stresses. They also participate in regulation of cell cycle, apoptosis, signal transduction, and other physiological processes. In fact, the wide roles of HSPs are based on the chaperon functions of stabilization multiple substrates that participate in almost every cell process.

### 4.1 Proteostasis network of HSPs

Protein synthesis and elimination are a dynamic cellular equilibrium process. In the proteome of mammalian cells, there are over10,000 proteins expressed, making it critical for proteostasis and cell survival to maintain protein stability and remove the aberrant proteins in the crowded cell (protein concentration > 300 g/L).^2^ The folding of nascent proteins to their native conformation requires the assistance of HSP chaperon network. In this process, nascent proteins are synthesized on ribosome as linear chains, which must then be folded into correct conformations to execute their functions. For proteins with less than 100 amino acids, they can be folded to native state spontaneously and rapidly.^86^ But the folding of larger proteins (>100 aa) or multidomain proteins are inefficient in cells and hampered by the increased number of folding intermediates or partially folded proteins^87^ as well as tendency for protein aggregation upon exposure to solvent in the hydrophobic region of partially folded proteins.^88^, ^89^

Both prokaryotic and eukaryotic organisms share conserved molecular chaperons to keep the orchestrate of the proteome stability. HSP70, HSP90, and HSP60 families act as foldases and participate in the de novo folding of nascent proteins, and as holdases—the large and small HSPs, preventing the proteins from aggregation. During translation on ribosome, ribosome‐binding chaperons interact with the nascent peptides. Trigger factor^90^ in bacteria first integrates with nascent peptide chain to assist the folding of 70% proteins,^90^, ^91^ and then the unfolded proteins are passed to the hub of folding network‐DnaK, which is the abundant DnaK‐DnaJ complex and fold about 20% proteins in bacteria proteome. The folding of the remaining 10% unfolded proteins to native conformation is accomplished on the GrpE fold machine. In eukaryotes, rather than the prokaryotic TF, nascent chain‐associated complex (NAC) and ribosome‐associated complex (RAC) associate with nascent polypeptides once they are synthesized on ribosomes.^92^, ^93^ The unfolded proteins are then delivered to HSP70‐HSP40, which work as an intersection before downstream folding machineries. On the one hand, HSP70 assists the folding of a fraction of proteins to native state. On the other hand, HSP70 connects the downstream folding chaperons and delivers the partially folded intermedia to HSP90 or TRiC chaperons. Some proteins in eukaryotes are transferred directly from HSP70 to HSP90 system by cofactor HOP for further folding,^94^ including kinases important for cell signaling transduction.^57^ By interacting with HSP90, the kinases are structurally stabilized and active in functions.^95^ The complicate chaperone mechanism is lack of structure basis due to the transient and dynamic nature of client proteins folding process and the client–chaperon complex structure is hard to be solved. Recently, using GR as client model, the Agard group reported the chaperon complex structure with client protein loaded by cryoelectron microscopy. The structure of Hsp90–Hsp70–Hop–GR complex describes the molecular basis of HSP90 chaperon cycles in coordination with HSP70,^96^ and based on the Hsp90–p23–GR structures, the complete remodeling cycle of client by chaperons was described for the first time.^97^

The protein folding pathways in bacteria and eukaryotes suggest that the coordination of protein folding relies on the different recognition modes of HSPs. HSP70 shows plasticity and promiscuity in substrate binding by recognizing the hydrophobic core motif.^38^ Therefore, HSP70 can participate in the folding of most of the intracellular proteins once they are synthesized and their hydrophobic residues exposed. TRiC recognizes a subset of domain‐specific folding intermediates folded by HSP70. Meanwhile, HSP90 recognizes substrates based on thermal and conformational stability.^57^

**FIGURE 2:**
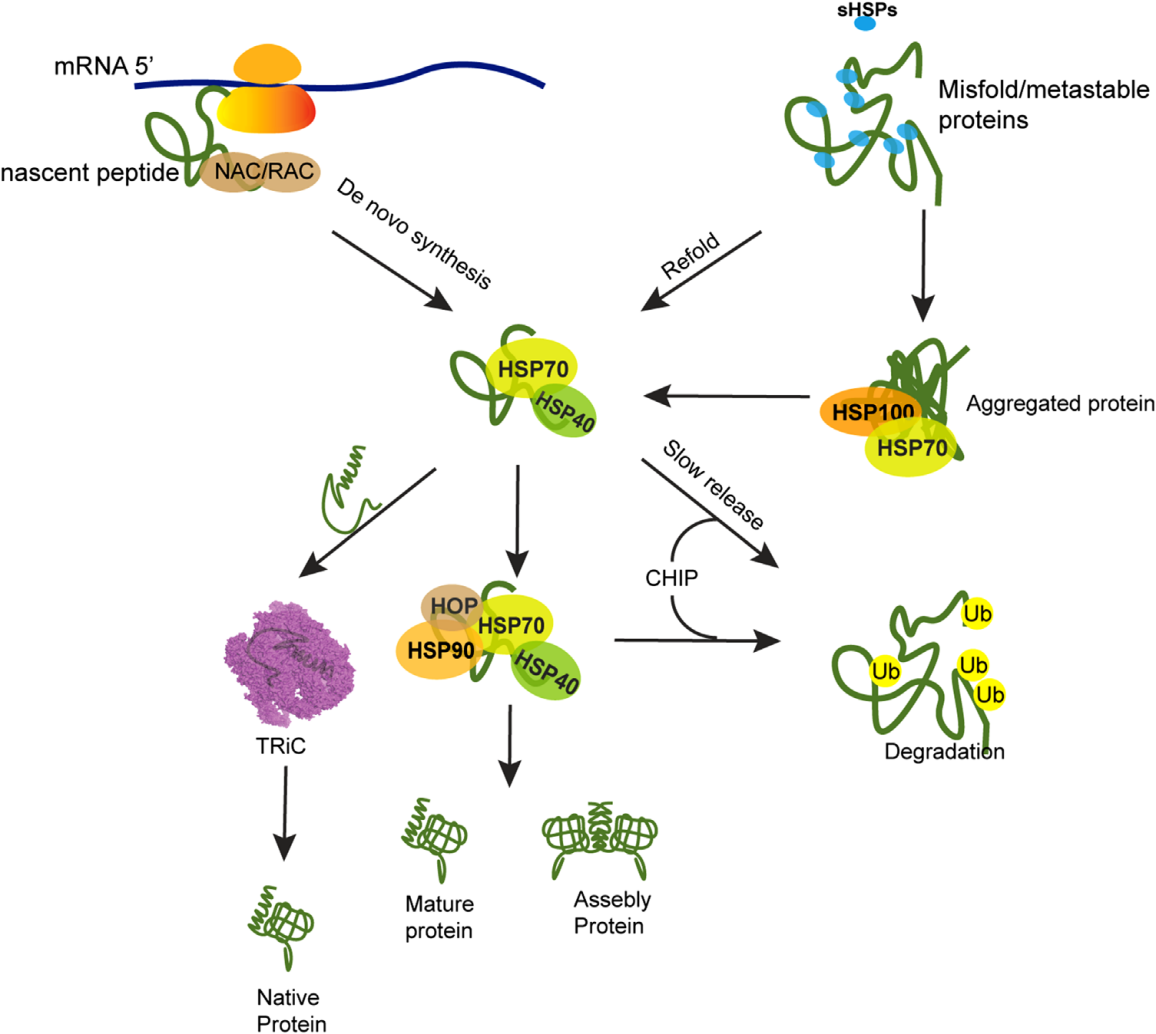
The proteostasis network of heat shock proteins. The heat shock proteins participate in the folding of de novo synthesized peptides, also they hold the metastable proteins and folding intermediates, preventing them from aggregation. In addition, HSPs and their cochaperones also take charge of protein quality control and connect with protein degradation pathways

Besides the folding of de novo proteins, the denatured proteins induced by stress or misfolded proteins are recognized by the HSP70 system for the protein triage decision, and some proteins can then be refolded to native state by HSP70 with assistance of HSP90.^5^, ^6^ However, the irreversibly misfolded proteins are transferred to downstream degradation machineries via either ubiquitin proteasome pathway or autophagy by coupling with multiple cochaperones. During proteasome degradation, CHIP, an E3 ligase, induces ubiquitylation of misfolded proteins and initiates proteolysis through proteasome by interact with EEVD domain of HSP70/90 and the a‐helical lid subdomain of HSP70 through the TPR domain.^49^ CHIP preferentially ubiquitinates the substrates that stay with Hsp70 longer than others. Besides CHIP‐mediated degradation, BAG1, the NEF of HSP70, also induces protein degradation through TRC8 E3 ligase.^98^ Meanwhile, BAG3 mediates degradation by autophagy and lysosome pathways (Figure 2).^44^, ^45^, ^46^

### 4.2 Stress response

Besides housekeeping functions, HSPs are also important for cells to cope with various stresses. In response to stresses, such as heat, hypoxia, oxidative stress, DNA damage, or accumulation of misfolded proteins, heat shock response (HSR) rapidly triggers the upregulation of multiple HSPs to buffer the disordered cellular environment and proteostasis, restoring cell structure and cellular metabolism.^99^ The protein family of HSF acts as stress sensors in cells.^7^ Under normal conditions, they are inactive in complexes with HSPs. Upon stress, they are released from the HSP‐HSF complexes, allowing HSF to oligomerize, translocate to nucleus, and bind to the promoter regions of multiple HSPs, triggering their rapid transcription.^99^, ^100^ However, not all the HSPs are regulated by HSF at the transcription level. Posttranscription and translational regulations are also key mechanisms for HSP expression. Under stressed conditions, 5′UTR methylation of stress‐induced transcripts are preserved by m6A “reader” YTHDF2, which, in turn, initiates the translation of HSPs in cap‐independent way, providing a novel regulation mechanism for stress induced mRNA translation.^101^

In stress conditions, cell death may be executed through both the intrinsic and extrinsic apoptosis. HSPs play dual roles in the regulation cell apoptosis with complicated mechanisms. In the intrinsic pathway, HSP70 inhibits the cytochrome c release by forming Bax‐HSP70‐HSP40 complex attenuating Bax mitochondria translocation. Meanwhile, HSPs can also regulate apoptosis through their canonical chaperon activity. For example, HSP90 and HSP70 can attenuate apoptosis through stabilizing and activating AKT signal pathway, which facilitates cancer cell survival.^102^ The inhibitor of apoptosis proteins (IAPs), such as c‐IAP1, XIAP, and cFLIPS/L (CFLAR), are also reported as obligate Hsp70 clients.^103^ By stabilizing IAPs, RIP1‐dependent apoptotic and necroptotic cascades are suppressed by HSP70.^104^ It has been recently reported that Bim act as HSP70 cochaperon and enhances the ATPase and oncogenic protein chaperon activities of HSP70 in order to prevent apoptosis in cancer cells.^105^ In addition, BCR‐ABL fusion protein induces the interaction of Hsp70 with Bim to prevent apoptosis in BCR‐ABL positive cells.^106^ Downstream of the mitochondria apoptosis pathway, direct HSP70‐Apaf1 interaction and HSP27‐cytochrome c interaction prevent apoptosome formation, thus inhibiting caspase 3/9 activations and terminating the intrinsic apoptosis pathway.^107^, ^108^ HSPs also participate in the regulation of extrinsic apoptosis pathway. For example, HSP70 interacts with DR4/5 to suppress the formation of TRAIL‐mediated death inducing signaling complex (DISC).^109^ In some breast cancer cells, TRAIL‐ and TNFa‐induced extrinsic apoptosis is inhibited by HSPB2 through suppressing caspases‐8/10 activations, which then blocking downstream Bid cleavage and the activation of caspase‐3.^110^

Although most HSPs are prosurvival, Hsp60 exhibits bidirectional apoptosis regulatory function through several mechanisms. They chaperon with anti‐apoptosis proteins Bcl‐2 and Bcl‐xl to attenuate the apoptosis,^111^ meanwhile stabilizing the survivin and CypD, which increase the cryoprotection and maintain the mitochondrial permeability.^68^, ^112^ In addition, HSP60 also suppresses the P53‐dependent apoptosis.^68^ In contrast, in the presence of certain pathological factors, HSP60 can also exhibit proapoptosis ability.^82^ Exogenous HSP60 is found to induce apoptosis through TLR4‐NF‐KB‐Caspase signal pathway in certain disease states.^113^, ^114^ Additionally, some apoptosis inducers such as staurosporine, disrupt the procaspase3–HSP60 complex, making caspase3 easier to be activated by cytochrome c, and this process is coupled with the release of HSP60 into cytosol.^115^

Due to the comprehensive functions of HSPs in protecting cell integrity and hemostasis, it has been found that multiple cancers show chaperon addiction and take advantages of HSPs to maintain the oncogenic protein's structure and activity, promote cancer cell survival, evade from the immune system, attenuate apoptosis, and develop drug resistance.^116^ Therefore, HSPs emerge as potential therapeutic targets in a variety of diseases.

## 5 HEAT SHOCK PROTEINS IN DISEASES

### 5.1 The role of heat shock proteins in cancers

In many malignancies, HSPs are frequently overexpressed and linked with poor prognosis, including lung cancers, gastric cancers, breast cancers, glioblastoma, PCs, and so on. HSPs have been reported associated with cancer cell proliferation, metastasis, and invasion. Thus, HSPs are effective biomarkers for cancer therapy (Table 2).

**TABLE 2:** Role of heat shock proteins in cancers

Name	Cancer type	Effects	References
HSP27	Prostate cancer	HSP27 is required for EGF‐mediated EMT via modulation of the β‐catenin/Slug signaling pathway in prostate cancer.	^121^
		HSP27 plays an important role in prostate cancer cell motility and metastatic progression	^138^
		HSP27 confers resistance to androgen ablation and chemotherapy in prostate cancer cells through eIF4E	^139^
	Colorectal Cancer	HSP27 is a key mediator in the progression and metastasis of CRC by regulating the store‐operated calcium entry.	^140^
		The elevated expression of HSP27 protein is a frequent event during the progression of CRC.	^141^
	Lung cancer	Increased HSP27 correlates with malignant biological behavior of nonsmall‐cell lung cancer and predicts patient's survival.	^142^
		Chemoresistance of lung cancer stem like cells depends on activation of HSP27	^143^
	Breast cancer	Phosphorylation of Ser78 of HSP27 is correlated with HER‐2/neu status and lymph node positivity in breast cancer	^144^
		HSP27 induces SUMOylation of HSPB8 to promote HSPB8 expression, thereby endorsing proliferation and metastasis of breast cancer cells.	^145^
		HSP27 is associated with decreased survival in node‐negative breast cancer patients.	^146^
HSP47	Colorectal cancer	HSP47 promotes tumor survival and therapy resistance by modulating AKT signaling via PHLPP1 in colorectal cancer.	^147^
		HSP47 is a predictive marker for lymph node metastasis in patients with colorectal cancer.	^148^
	Prostate cancer	HSP47 confers chemoresistance on pancreatic cancer cells by interacting with calreticulin and IRE1α.	^149^
	Breast cancer	HSP47 promotes metastasis of breast cancer by interacting with myosin IIA via the unfolded protein response transducer IRE1α.	^150^
	Lung cancer	HSP47 promotes cell migration and invasion through AKT signal in nonsmall‐cell lung cancer.	^151^
	Glioblastoma	HSP47 plays an important role in GBM tumor formation, invasion, and angiogenesis.	^126^
	Head and neck cancers	HSP47 is associated with the prognosis of laryngeal squamous cell carcinoma by inhibiting cell viability and invasion and promoting apoptosis.	^152^
HSP60	Hepatocellular carcinoma	HSP60 exerts a tumor suppressor function by inducing cell differentiation and inhibiting invasion in hepatocellular carcinoma.	^153^
	Colorectal Cancer	High HSP60 expression is important for CRC progression.	^154^
	Gastric Cancer	HSP60 overexpression is associated with the progression and prognosis in gastric cancer.	^155^
	Prostate cancer	HSP60 expression is strongly associated with prostate cancer lymph node metastasis.	^122^
HSP70	Leukemia	HSP70 expression is increased in the blood cells of patients with ALL, and inhibiting HSP70 could suppress cell proliferation and induce apoptosis.	^156^
		HSP70 is involved in the progression of FLT3‐ITD‐positive AML.	^119^
	Breast cancer	High HSP70 expression is found to be related with breast cancer lymph node metastasis.	^127^
		HSP70 is overexpressed in breast cancer.	^129^
	Lung cancer	HSP70 promotes SUMO of HIF‐1α and promotes lung cancer invasion and metastasis.	^157^
	Prostate cancer	HSP70 binds to the N‐terminal domain of androgen receptor and modulates the receptor function in prostate cancer cells.	^158^
	Colorectal Cancer	HSP70 overexpression can predict poor survival in colorectal cancer patients.	^159^
	Head and neck cancers	HSP70 is a potential biomarker for detecting tumors and for monitoring the clinical outcome of radiotherapy in SCCHN patients.	^160^
	Glioblastoma	HSP70 promotes survival of C6 and U87 glioma cells by inhibition of ATF5 degradation.	^161^
HSP90	Colorectal cancer	HSP90 plays an important role in promoting EMT transition, migration, and invasion in colorectal cancer.	^132^
		HSP90 is needed to cooperate with CD24 to enhance STAT3‐mediated VEGF transcription to inducing colorectal cancer angiogenesis.	^135^
	Leukemia	HSP90 acts as the molecular chaperone and is highly expressed in several therapy‐resistant leukemia subtypes, thereby ensuring correct protein folding of several oncogenic proteins such as BCR‐ABL1 and FLT3‐ITD.	^120^, ^162^
	Prostate cancer	HSP90 plays an important role in advanced prostate cancer growth and survival.	^163^
	Lung cancer	High HSP90 expression correlates with poorer overall survival in lung cancer patients.	^164^
	Ovarian cancer	HSP90 interaction with Lamin‐A is required for DNA damage repair and chemoresistance of ovarian cancer cells.	^165^
	Breast cancer	Elevated HSP90 expression in breast ductal carcinomas contributes to the proliferative activity of breast cancer cells.	^166^

Leukemia is a hematological neoplasm that affects blood and bone marrow. Both acute myeloid leukemia (AML) and acute lymphoblastic leukemia (ALL) cells were shown to express high levels of HSPs. Patients with lower HSP expression usually have a higher rate of complete remission (CR) and their overall survival (OS) was considerably longer.^117^ For example, FLT3 is the most frequently mutated gene in human AML^118^ and HSP70 plays an important role in the progression of FLT3‐ITD‐positive AML. Knocking down inducible HSP70 was enough to destabilized FLT3‐ITD protein and inhibit cell proliferation and tumor growth in FLT3‐ITD‐positive AML cells. Thus, for FLT3‐ITD‐positive AML, targeting HSP70 could be a feasible treatment strategy.^119^ p210(BCR‐ABL) or p185(BCR‐ABL) fusion is an oncogenic driving force in chronic myelogenous leukemia (CML). ABL tyrosine kinase is one of the HSP90 clients implicated in the development of chronic myeloid leukemia (CML). When the interaction between BCR‐ABL and HSP90 is disrupted, the cellular level of BCR‐ABL protein is reduced and the BCR‐ABL1‐STAT5 signaling pathway is thus inhibited,^120^ suggesting that HSP90 appears to play an essential role in BCR‐ABL mutant leukemia. PC is the world's second most frequent malignancy in men. Metastasis is a great challenge for PC and it can be not cured by chemotherapy and ionizing radiation. In PCs, HSP27 has been reported as a key regulator of Hippo pathway, and the upregulation of HSP27 activates oncogenic and metastatic pathways by increasing the nuclear localization of transcription factor YAP. Besides, HSP 27 is also essential for EGF‐mediated EMT via β‐catenin /slug signaling pathway.^121^ In poorly differentiated PC cells, HSP 60 protein is often overexpressed, which is strongly associated with lymph node metastasis.^122^ Increased level of HSP 60 expression is related to poor survival of PC patients. Castration‐resistant PC (CRPC) is dependent on HSP60 through moderate mitochondrial spare respiratory capacity.^123^

Glioblastoma multiforme (GBM) is an aggressive and lethal brain tumor in adults.^124^ In GBM, mTORC1 signaling is hyperactivated and promotes growth and proliferation, and thus could be a promising therapeutic target in GBM. Downregulation of HSP60 enhances the ROS generation, resulting in AMPK pathway activation. The activated AMPK reduces protein translation by suppressing mTORC1‐mediated S6K and 4EBP1 phosphorylation, inhibiting cancer cell proliferation.^125^ HSP47 has been reported to be highly overexpressed in GBM and is associated with tumor grade. HSP47 is involved in tumor development, invasion, and angiogenesis. The high expression of HSP47 in GBM cell lines leads to an increase in CD44+ cells, promoting the survival of GBM stem like cells by modifying the extracellular matrix of the tumor microenvironment (TME) via the TGF pathway.^126^

Breast cancer is the main cause of cancer death among women worldwide. With the rapid drug discovery in the past few decades, a variety of drugs with different action mechanisms have improved the prognosis for breast cancer patients. However, metastasis is still a great challenge for cancer therapy. In breast cancer models, high HSP70 expression is found to be related with lymph node metastasis.^127^ The epithelial‐mesenchymal transition (EMT) is a developmental process that permits stationary epithelial cells to migrate and invade as single cells. EMT plays an important role in prompting cell metastasis.^128^ Inhibiting HSP70 have been shown to reduce invasion and migration by downregulating EMT pathway through modulating the expression of proteins involved in EMT such as N‐Cadherin, P‐Cadherin, Vimentin, and so on.^129^ Furthermore, HSP70, together with HSP90, activates matrix metalloproteinase 2 (MMP‐2), thereby increasing the migration and invasion of breast cancer cells.^130^

The most common cancer‐related cause of mortality is colorectal cancer (CRC), and metastasis is the major cause of death in CRC patients.^131^ The nuclear factor kappa B (NF‐kB) and hypoxia‐inducible factor‐1 (HIF‐1a) are crucial in modulating CRC metastasis, both of which are client proteins of HSP90. The protein HSP90 has been linked to the promotion of EMT transition, migration, and invasion in CRC s.^132^ In addition, angiogenesis also plays a significant role in tumor progression, including CRC.^133^ Overexpression of CD24 is common in colorectal malignancies, and HSP90 is required for enhancing STAT3‐mediated VEGF transcription and initiating CRC angiogenesis.^134^ Inhibiting HSP90 shows great efficiency in blocking CD24‐mediated CRC angiogenesis.^135^ Besides, HSP110 is highly expressed in CRC and plays a role in the proliferation of CRC through activating STAT3 and promoting its transcriptional function.^136^ Also, HSP110 is related to nodal metastasis that is a predictive marker for CRC metastasis.^137^

### 5.2 Roles in neurodegeneration diseases

Alzheimer's disease (AD) is the most prevalent neurodegenerative illness, characterized by aberrant accumulations of hyperphosphorylated tau protein in brain cells, resulting in neurofibrillary tangles (NFTs) and improper folding of amyloid‐peptides (Aβ).^167^ According to research, HSP not only plays a vital function in tau protein accumulation/degradation, but it also prevents Aβ‐related toxicity. HSP90/HSP70 is important in maintaining the normal physiological state of Tau protein as well as blocking aberrant phosphorylation and accumulation of Tau protein, and involved in the pathologic process of the AD‐associated Tau protein and Aβ.^168^, ^169^, ^170^ Tau protein serves as a client for HSP90/HSP70 that can regulate Tau protein metabolism. Many studies have shown that inhibiting HSP 90 could reduce phosphorylation of Tau through proteasomal degradation.^171^ HSP70 maintains tau and Aβ homeostasis through three mechanisms: inhibiting tau and Aβ aggregation, mediating tau return to microtubules, and accelerating Aβ clearance.^172^ Many neurodegenerative disorders, such as AD, Parkinson's disease, and Huntington's disease, have been linked to mitochondrial malfunction.^173^ Multiple aspects of mitochondrial function are hampered by Aβ, including the creation of ROS, the disfunction in energy metabolism, and so on.^174^ It has been reported that Aβ can decrease the activity of complex IV and HSP60 can eliminate this effect of Aβ. One of the reasons is that HSP60 can inhibit Aβ amyloid peptide aggregation by disrupting fibrillogenesis. Despite the fact that HSP60 has been shown to have a neuroprotective role in AD, many studies demonstrate that upregulation of HSP60 expression can worsen the disease through triggering the release of inflammatory cytokines and accelerating neuronal cell death by binding to TLR4.^175^ Parkinson's disease is distinguished by the accumulation of cellular misfolded α‐Syn protein and the death of dopaminergic neurons in the brain. HSPs are the most effective and well‐preserved cellular defense system in neurons, maintaining α‐Syn protein homeostasis by correcting protein folding, refolding partially misfolded proteins, and destroying potentially harmful aggregates.^176^, ^177^ HSPs also play a role in the chaperone‐mediated autophagy apparatus and inhibiting α‐Syn aggregation by the UPS.^176^, ^178^, ^179^

### 5.3 HSPs in cardiovascular diseases

CVDs are a group of complex pathophysiological processes that occur in the heart or blood vessels, such as hypertension, atherosclerosis, coronary artery disease, arrhythmias, heart failure, and idiopathic LV heart dysfunction. Despite a variety of advanced treatments available, CVDs remain a leading cause of morbidity and mortality worldwide and also a major cause of disease burden in the world.^180^, ^181^ And HSP involves in normal physiological pathological functions of cells, especially in CVDs.

HSPs are immunodominant molecules that stimulate both innate and disease‐related immune responses. Inflammation causes cardiovascular dysfunction by causing cellular stress events such as apoptosis, oxidative and shear stress, as well as cellular and humoral immune responses, all of which impair the system's structure and function^182^ (Table 3).

**TABLE 3:** Heat shock proteins involvement in cardiovascular diseases

Superfamily	Protein names	Clinical Manifestations	References
sHSPs	HSPB1	Coronary artery disease	^183^
	HSPB2	protective effects against heart diseases such as cardiac hypertrophy and ischemia	^184^
	HSPB3	Deregulation of myoblast viability	^185^
	HSPB5	Desmin‐related myopathy	^186^, ^187^
	HSPB6	Decreased contractile function	^188^
	HSPB7	No protection against age‐related dysfunction	^189^
	HSPB8	Lack of protection after myocardial ischemia	^190^, ^191^
HSP40	DNAJA3	Respiratory chain deficiency	^191^, ^192^
HSP60	HSP60	Protection from atherosclerosis and proliferation of vascular smooth muscle cells	^193^
HSP70	HSPA1A	during myocardial ischemia/reperfusion HSP70 had protective effects	^194^
	HSPA8		
HSP90	HSPC4	Increased in plaque stability Cardiomyocyte apoptosis	^195^

In CVDs, HSPs are a double‐edged sword.^196^ On the one hand, HSPs are initially induced by stress stimuli. HSPs are expressed pronouncedly in CVDs, which play a cardioprotective role by inhibiting apoptosis.^197^ Numerous studies have shown that preinduction of HSPs by mild stress has a protective effect on a more severe stress, which is related to the amount of induced HSP.^198^ In addition, overexpression of HSPs in cultured cardiomyocytes or transgenic animals or intact hearts using viral vectors also has a protective effect, which directly demonstrates the ability of HSPs to protect cardiac function.^198^ On the other hand, HSPs and autoimmune responses directed against them may be involved in the pathogenesis of atherosclerosis. For example, the expression of host‐protected HSP60 (hHSP60) on vascular endothelial cells may be the target of cross‐reactive autoimmune responses. Thus, there may be a cross‐reaction to the immune response of bacterial HSPs during infection due to the high sequence consistency between human and microbial HSPs. GroEL has high immunogenicity and cross‐reacts with hHSP60 expressed on endothelial cells, leading to endothelial dysfunction and atherosclerosis. This triggers an inflammatory cascade and accelerates the progression of atherosclerosis. Early fatty streak lesions may progress to atherosclerotic plaque formation if infections and cardiovascular risk factors persist. Under normal physiologic conditions, HSPs play a protective role in the arterial wall. However, due to their highly conserved sequences, HSPs expressed on the vascular endothelial cell surface can act as targets for detrimental autoimmunity in disease states.^199^

Variety of HSPBs including HSPB1, HSPB2, HSPB3, HSPB5, HSPB6, HSPB7, and HSPB8 express in cardiac and skeletal muscle cells. These family members are generally considered as the focus of CVDs researches and the latest evidence highlights their role in cardiovascular protection. HSPB1 is a widely expressed multifunctional protein chaperone. Compared to healthy subjects, HSPB1 secretion decreased in human atherosclerotic plaques and HSPB1 levels are reduced in plasma. High expression of HSPB2 in heart has protective roles on some heart diseases such as cardiac hypertrophy and ischemia.^200^ HSP70 also plays a role in the development of hypertension. Due to the relationship between genetic polymorphisms of HSP70 and essential hypertension, HSP70 level is elevated in hypertensive patients’ circulation and kidney.^201^

There is a modulation between chaperones and cochaperones during the development of CVDs. Several CVDs occur due to out of physiological balance between protein synthesis, folding, and degradation resulting in accumulation of misfolded proteins. It is worth mentioning that different HSP proteins have opposite effect in promotion or inhibition of CVDs as well as related symptoms. Therefore, it is challenged to develop therapeutic strategies for CVDs based on HSPs that need balance their physiologic and pathologic roles. Better understanding of the relationship between HSPs and CVDs may speed up the development of new therapeutic strategies.^202^

### 5.4 HSPs in autoimmune diseases

**FIGURE 3:**
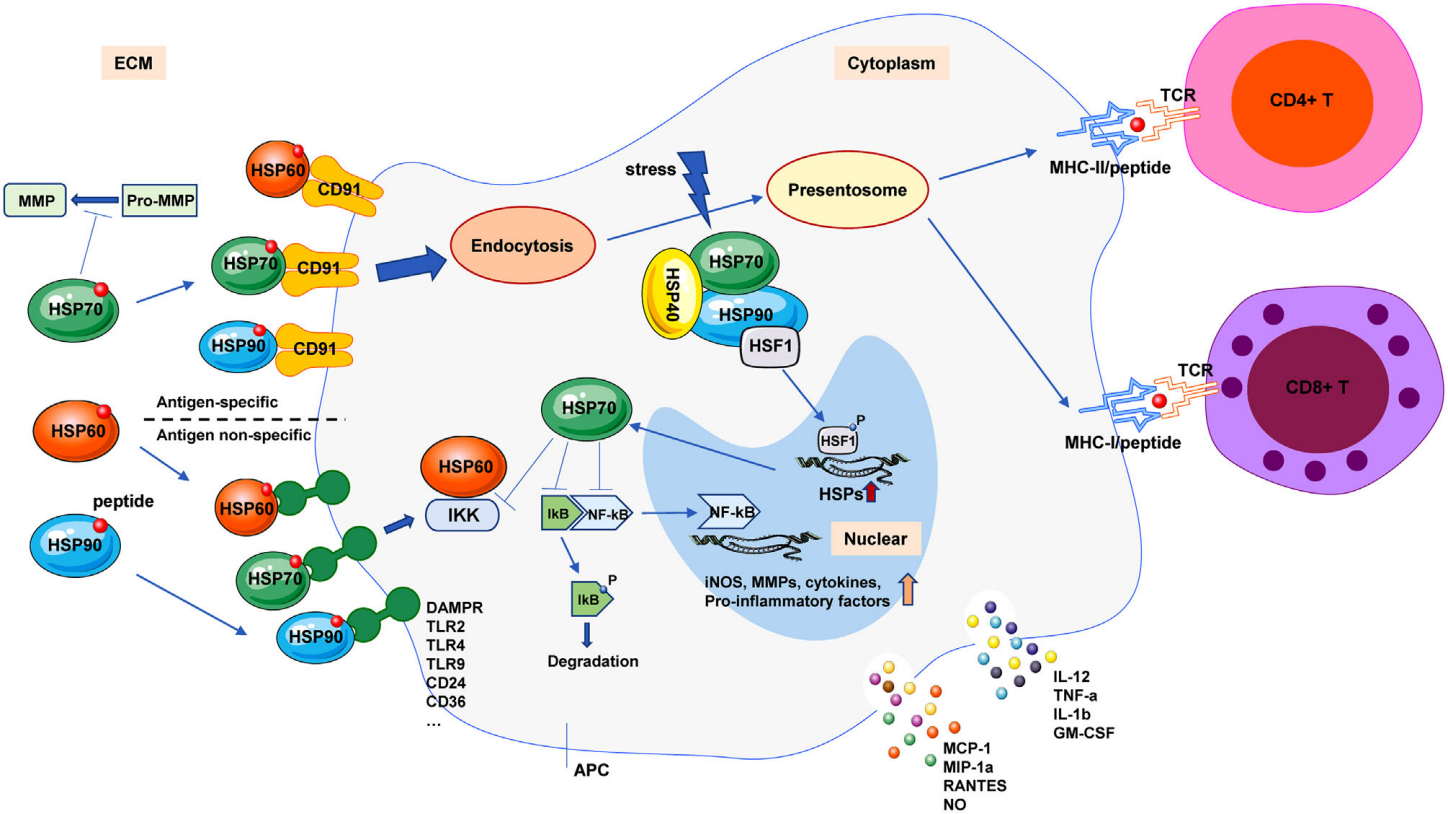
The HSP‐APC interaction modulates innate and adaptive immune responses

As we known, immune responses could be enabled by danger‐associated molecular pattern (DAMP), which is released by damaged or stressed tissues. HSPs were originally incorrectly classified as DAMPs, because HSPs are a group of constitutive and/or stress‐induced proteins, including thermal stress, infectious agents, intracellular stress, and so on.^203^ So far, the immune response to HSPs has widely been studied in various inflammatory and autoimmune diseases with either immunoregulatory or immunostimulatory responses (Figure 3). Their bimodal and sometimes paradoxical roles in autoimmune diseases are outlined as follows.

#### 5.4.1 Small HSP in autoimmune diseases

Apart from chaperoning functions, the small HSPs also play an important role in cytoskeletal organization responding to cellular stress.^204^ Among them, the most studied sHSP is HSP27. Aberrant phosphorylation of HSP27 was often increased in various autoimmune diseases, such as autoimmune skin diseases (pemphigus vulgaris and pemphigus foliaceus)^205^ and myasthenia gravis (MG).^206^ Anti‐HSP27 antibody levels were remarkably higher in broncho‐alveolar lavage (BAL) in patients with bronchiolitis obliterans (BOS) compared to lung transplant recipients without BOS.^207^ HSP27 serum autoantibodies also correlate to Glaucoma (increased intraocular pressure)^208^ and Guillain Barret syndromes.^209^ NZBxW/F1 mice is a well‐established spontaneous lupus nephritis model. HSP27 could induce mesangial cell activation, which has a pivotal role in lupus phenotype development.^210^ However, HSP27 can also exert anti‐inflammatory effect, as reduction of HSP27 leads to higher expression of pro‐IL‐1β and significantly more IL‐1β releasement in LPS‐treated monocytes.^211^ In an ischemic acute kidney injury (AKI) mouse model, HSP27 was reported to play a protective role in kidney injury and neutrophil infiltration.^212^

#### 5.4.2 HSP40 in autoimmune diseases

HSP40 often exerts a pro‐inflammatory function in autoimmune diseases. HSP40 is reported as a biomarker of fibrillary glomerulonephritis, which is an autoimmune disease characterized by the extracellular deposition of nonamyloid fibrils.^213^ DNAJB9, a member in HSP40 family, colocalizes with the fibrils,^214^ indicating that HSP40 causes an immunostimulatory response in this context. In atherosclerosis, HDJ‐2, a homolog of human HSP40 found in *E. coli*, is highly expressed in atheromatous lesions, which may play an important role in T‐cell activation in the development of atherosclerosis.^215^ HSP40/HSP70 expression is increased in stoke patients, which may lead to autoimmune responses against these HSPs.^216^ Bullous pemphigoid, a bullous autoimmune disease, is characterized by the presence of autoantibodies against components of the dermal–epidermal junction. Compared to healthy control, circulating anti‐HSP40 autoantibodies were elevated in these patients.^217^ Furthermore, HSP40 was found able to stimulate RAW264.7 cells to secrete IL‐6 by inducing PI3K/JNK signaling and inflammation.^218^

However, HSP40 was responsible for the decline of pro‐inflammatory cytokine TNFα and the increase of anti‐inflammatory cytokine IL‐10 in the synovial fluid of juvenile idiopathic arthritis patients. Further study showed that the exposure of synovial fluid monocytes (SFMCs) to HSP40 peptide fragments induced Treg cells (CD4^+^, CD25^high^) with an increase of FoxP3, IL‐10, and CTLA‐4 mRNA, which may reverse the ongoing inflammation of the disease.^219^

#### 5.4.3 HSP60 in autoimmune diseases

HSP60 is mainly expressed in mammalian cells, whereas HSP65 is predominantly expressed in nonmammalian cells, like Mycobacterium.^220^ HSP60/65 are HLA‐DR binders and able to present client peptides easily to antigen‐presenting cells (APCs). Self HSP60 undergoes a complete antigen processing within APCs and induces Th2 responses and tolerogenicity. On the other side, nonself HSP65 undergoes an incomplete antigen processing within APCs and induces Th1 responses and autoimmunity.^221^, ^222^ Molecular mimicry between human HSP60 and HSP65 could induce autoimmune phenomena. So, anti‐HSP60/65 autoantibodies were detected parallelly in autoimmune diseases, including SLE, Sjögren syndrome, undifferentiated connective tissue disease,^223^, ^224^, ^225^ rheumatoid arthritis,^209^, ^226^, ^227^ and autoimmune hepatitis.^228^

Type I diabetes mellitus (T1D) is an autoimmune disease, and streptozotocin‐induced diabetes (STZ model) and nonobese diabetic^229^ mice are two typical animal models for T1D. On the one hand, HSP60‐induced T‐cell activation and anti‐HSP60 antibody could be detected in experimental models of T1D, which contributes to diabetes aggravation.^230^ On the other hand, HSP60 elicited a Th2 response and inhibited diabetes progression in an experimental STZ model. Immunization with mycobacterial HSP65 has also been found to prevent disease aggravation in NOD mice.^231^ HSP60‐p277 peptide immunization in NOD mice induced a Th2 response with more IL‐10 and IL‐4 releasement, which was accompanied by Th1 response downregulation.^232^

In a rat arthritis model, HSP60 administration showed increased number of Treg cells (CD4+, FoxP3+) in the joint‐draining lymph nodes and improved arthritis symptoms. HSP60 could induce Treg cell proliferation mediated by TLR9, leading to IL‐10 production.^233^ Different domains of HSP65 exert different actions in autoimmune arthritis, and HSP65 P118‐388 causes Treg cells expansion and reduces autoimmune arthritis, whereas HSP65 P180‐188 does not.^234^ Besides, immunization with HSP60/65 decreased autoantibodies against erythrocytes in mouse model of hemolytic anemia,^234^ but increased inflammatory responses in mouse models of atherosclerosis^235^ and intestinal autoimmune disease.^224^

Interestingly, TLRs appear to play a key role in the immune regulation of HSP60/65. When HSP60/65 binds to TLR9/HLA‐DR, they induce a Th2 cytokine response in APCs. Otherwise, when HSP60/65 binds to TLR2/HLA‐DR, they induce a Th1 cytokine response in APCs.^221^, ^236^

#### 5.4.4 HSP70 in autoimmune diseases

HSP70 sometimes induces an immunostimulatory response in autoimmune diseases by stimulating APCs to secrete more pro‐inflammatory cytokines, such as IL‐12, TNFα, and IL1β, and facilitate the maturation of immature dendritic cells.^237^, ^238^, ^239^ HSP70/Ro52 and HSP70/Ro53 complexes were reported to induce more macrophages and cytotoxic T cells infiltration.^240^ HSP70 serum autoantibodies were elevated in patients of thyroiditis,^241^ inner ear disease,^242^ and diabetic microangiopathy,^243^ thereby involved in immune response modulation. In mouse model of salt‐sensitive hypertension, circulating anti‐HSP70 antibody was related to increased renal inflammatory infiltration.^244^ Recombinant HSP70 immunization in RIP‐GP/P14 mice could induce the onset of diabetes, suggesting an in vivo pro‐inflammatory action of HSP70 in autoimmunity.^239^

In most cases, HSP70s exert an immunoregulatory action in autoimmune diseases. Multiple HSP70 client peptides could bind to HLA‐DR and induce Treg cell expansion. In a mouse model of autoimmune arthritis, HSP70 treatment induced Treg cell expansion, elevated LAG3 expression in Treg cells, and increased IL‐10 production, resulting severity reduction of the disease.^245^ Additionally, HSP70‐HINT1 (histidine triad nucleotide‐binding protein‐1) downregulated immune response through CD94 and NKG2D signaling in an experimental autoimmune encephalomyelitis (EAE) model,^246^ suggesting that HSP70 expression was responsible for the reduced clinical inflammation scores in such models. In this context, inducible nitric oxide synthase (NOS)^247^ production, RANTES, and NF‐κB mRNA transcription were downregulated,^248^ indicating that HSP70 plays an anti‐inflammatory role in these autoimmune diseases.

#### 5.4.5 HSP90 in autoimmune diseases

HSP90 could exert either immunoregulation or immunostimulation effects upon the assembly of different receptors and signaling pathways. When binding with certain receptors (CD36, CD91, and TLR2/4) on APCs, HSP90 blocks CTL (CD8^+^ T) expansion and downregulates T‐cell responses; when binding with other receptors (CD25 and P2 × 7R), HSP90 promotes autoimmunity. HSP90 induces IL‐1β production through binding with ATP‐gated P2X cation channel receptor (P2 × 7R) on APCs. HSP90 can also induce macrophage activation through binding with CD25, and propagate T‐cell responses, which is then followed by anti‐HSP90 antibody production.^236^ HSP90 autoantibodies were detected in systemic lupus erythematosus and other autoimmune diseases.^227^ In mouse models of autoimmune exocrinopathy^249^ and anti‐collagen VII autoimmunity,^250^ HSP90 could induce the infiltration of inflammatory cells and promote autoimmunity. But in other mouse models of autoimmune diseases, sucg as EAE, T1D, and skin diseases, immunization with HSP90 could reduce autoimmunity.^251^, ^252^, ^253^ In a rat model of autoimmune arthritis, HSP90 reduced arthritis symptoms and induced immune tolerogenicity.^234^

**FIGURE 4:**
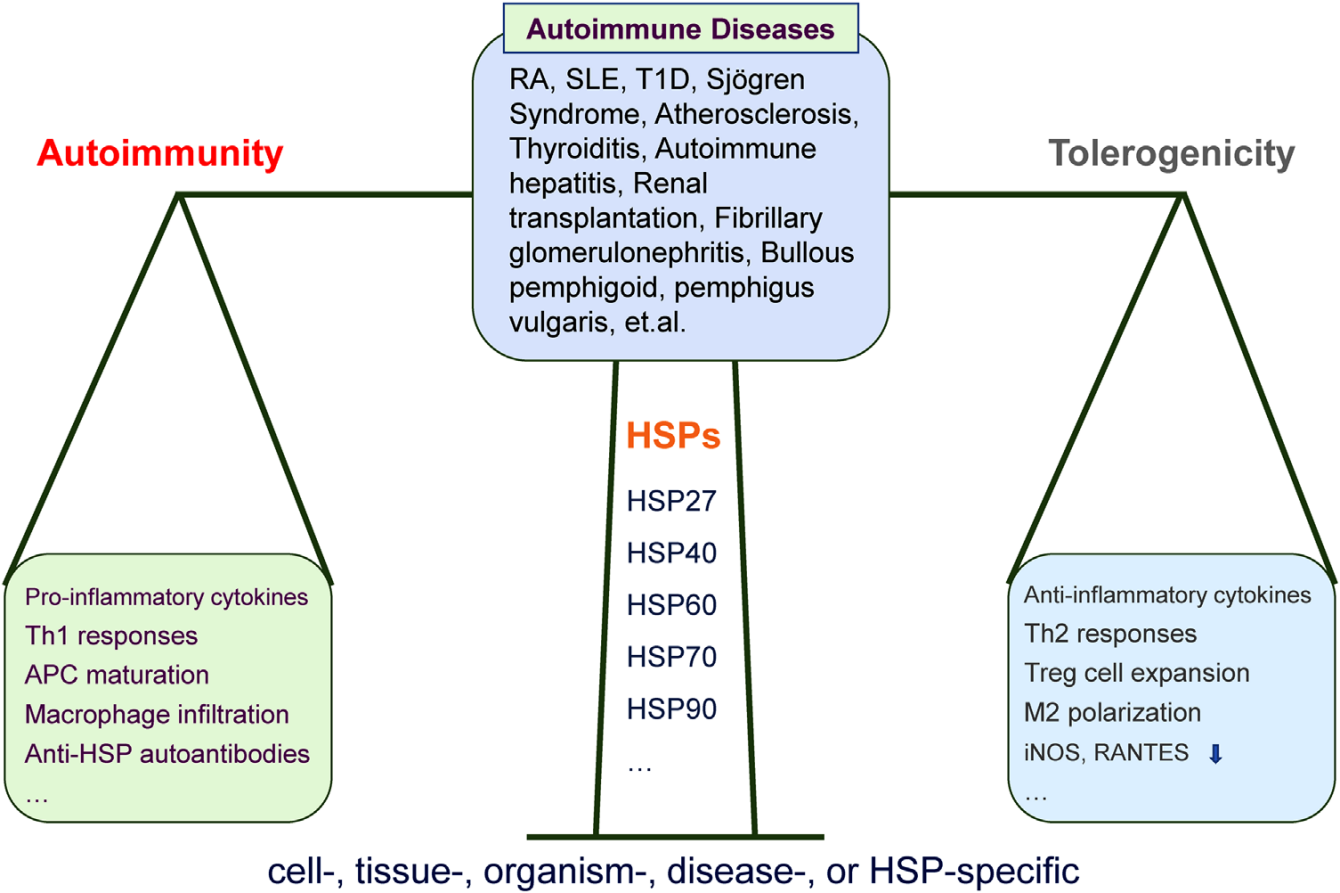
Immunomodulatory actions of HSPs in autoimmune diseases

Taken altogether, immune responses could be finely tuned by the functions of different HSPs, which work like a double‐edged sword. Different HSPs, in cooperation with different receptors (MHC‐II, TLR, etc.), play different immunomodulatory functions in autoimmune diseases, which could be cell‐, tissue‐, organism‐, disease‐, or HSP‐specific (Figure 4).

## 6 HSPSs AS POTENTIAL THERAPEUTIC TARGETS

Studies of the roles of HSPs in the cause and progression of other severe diseases, such as neurodegeneration, CVD, autoimmune disease, and infection, have made tremendous efforts aiming to identify novel therapies directly targeting HSPs.^254^ Therefore, HSPs, such as HSP90 and HSP70, have aroused great interest as potential therapeutic targets^255^, ^256^ (Table 4).

**TABLE 4:** HSPs as potential therapeutic targets in different types of diseases

Diseases	Therapeutic targets	Clients or mechanisms	References
Cancers	Oncogenic HSP90	Cell cycle regulation, immune responses Modulate aberrant protein: p53, HER2/ERBB2, AKT, BCR‐ABL, RAF1, and so on	^257^, ^258^
	HSP70	Affect variety apoptosis‐inducing pathways, DISC Tumor suppression pathways, lysosomal membranes, and so on	^259^
	Exosomal HSP60	Increased in patient tumor‐tissue samples or EXs from blood	^260^, ^261^, ^262^, ^263^
	HSPgp96	Multiple clients: HER2, integrins, TLRs, LRP6, and IGF	^264^, ^265^
	HSF1	Supporting of cell migration, invasion, proliferation, and cancer cell metabolism	^266^, ^267^, ^268^
	HSP27	Increased or low expression levels in different cancers or during chemotherapy	^269^, ^270^, ^271^, ^272^
	HSP40	Upregulated in different cancers and serum of cancer patients	^273^, ^274^, ^275^, ^276^
Neurodegeneration diseases	HSP90	p‐tau, p35, LRRK2, and so on	^277^, ^278^, ^279^, ^280^
	HSP70	α‐syn, Reducing loss of neurons	^281^, ^282^
	HSP27	Antioxidant activity Inhibiting cell death pathways Modulating tau dynamics Amyloid β (Aβ) peptides, α‐syn aggregation	^283^, ^284^, ^285^, ^286^, ^287^
Cardiovascular diseases	HSP22	NOS, protects mitochondrial function, Rho GTPase pathway, glycogen synthesis	^288^, ^289^, ^290^, ^291^
	HSP27	Angiotensin II, Nuclear factor‐B pathway, Cytokines	^292^, ^293^, ^294^, ^295^, ^296^, ^297^, ^298^, ^299^
	HSP60	Mechanism behind remains unclear	^300^, ^301^, ^302^, ^303^, ^304^
	HSP70	NO generation, Ca2+ channel, ATP‐sensitive potassium channels	^305^, ^306^, ^307^
Autoimmune diseases	HSP60	Stimulate macrophages, Reacted with various infectious microorganisms	^308^, ^309^, ^310^, ^311^
Inflammation	HSP90, HSP70	Production of proinflammatory cytokines, antigen presentation, and so on	^312^, ^313^, ^314^, ^315^
Infection diseases	HSP90	antifungal in morphogenesis	^316^
	HSP70	HIV in CD4+ T cells	^317^, ^318^
	HSPgp96	Hepatitis B virus	^319^
	HSP40	Stimulates Th1 and Th17 against Streptococcus pneumoniae	^320^

### 6.1 HSPs as targets in cancers

Since HSPs help proteins folding and function in cells, they are present at high levels in cancer cells where defective and mutant proteins are abundant.^321^ Blocking the activity HSP90 is already being explored in the treatment of cancers.^322^ Other HSPs, including HSP70 and gp96, are also being well studied in vaccines to treat cancers.^323^, ^324^

Among all the HSPs, HSP90 acquired the most interest and is the best understood during the drug discovery process.^325^ HSP90 is a ubiquitously expressed gene that is one of the most abundant intracellular proteins in mammalian cells. HSP90 plays a crucial role in the conformational maturation, protein stability, and function of oncogenic signaling, such as mutated p53, AKT, HER2/ERBB2, BCR‐ABL, and RAF1, as well as many other members that are important in cell cycle progression and immune responses, and it has been recognized as a potential therapeutic target for a number of diseases correlated with aberrant protein signaling, especially in cancers.^257^, ^258^ The interest in anticancer ability of HSP90 was first discovered during a compound screening for v‐Src transformed cells, in which the antibiotic geldanamycin (GM) emerged as a hit.^326^ The potential therapeutic benefit of the selective and potent anticancer activity of GM inspired researchers to identify its target. Later, GM was verified as an HSP90 inhibitor that targets the N‐terminal nucleotide binding pocket by different groups.^255^, ^327^ Subsequently, the mechanistic study uncovered the HSP90 substrate preference of mutant p53 over wild‐type p53, which explained the selectivity of GM and its derivative 17‐AAG through targeting a ubiquitous protein. Researchers further showed that, compared to normal cells, the HSP90 multichaperone complexes derived from tumor cells exhibited increased ATPase activity and showed higher affinity for HSP90 inhibitors, which provided a reasonable explanation for the selectivity observed with HSP90 inhibitors.^328^

However, cancer cells only harbor about 20–30% HSP90 of total, consisting of stressed HSP90 chaperones that are involved in oncogenic partners (e.g., cochaperones) to maintain the malignant phenotype, thereby also named as oncogenic HSP90. Inhibitors have varying ability to recognize the oncogenic HSP90 fraction.^329^ Moreover, studies have shown that posttranslational modifications (PTMs) may influence HSP90 chaperone activity and their therapeutic benefit in several ways, including affecting the affinity and hydrolysis of ATP, as well as the association with cochaperones and client proteins. The extents and types of PTMs in normal versus aberrant cells are also different and can impart drug selectivity towards cancer cells, providing a therapeutic rationale for HSP90 as anticancer targets.^330^ The development of HSP90 inhibitors is by far the most advanced, and there have been over 20 inhibitors entering into clinical evaluation for the treatment of cancers, suggesting the great therapeutic advantage of HSP90.^325^, ^331^

HSP70 is one of the cochaperones of HSP90, and it also cooperates with other chaperone systems including HSP60 and small HSPs. Together, they compose a dynamic and functionally versatile network in a broad range of cellular housekeeping activities and stress‐related responses. It is known that some members of the HSP70 families are abundantly present in cancer cells and provide malignant cells with selective advantages by diminishing apoptosis signaling and promoting metastasis that is associated with poor prognosis, thus making them potential drug targets.^256^, ^332^ For instance, genetic studies strongly suggest that HSP70 plays critical roles in tumor progression and metastasis, which may involve the downregulation of tumor suppression pathways.^333^ This direct involvement of HSP70 in cancer tightly associates tumor survival and growth with the HSP70 expression. HSP70s not only inhibit various apoptosis‐inducing pathways, but also block death‐inducing signaling complex (DISC) formation.^334^, ^335^ Moreover, studies have indicated that HSP70 stabilizes lysosomal membranes, allowing tumor cells to escape from cell death.^336^ Further research revealed that inactivation of HSP70 reduced tumor invasiveness and metastatic potential of different cancer cell lines (e.g., breast cancer).^259^ These explorations may give new clues for drug development in order to negatively modulate HSP70 activity for cancer treatment.

Blachere's group showed that HSP70‐specific peptide‐complex induced antigen‐specific CD8+ T cell response, suggesting that the immunogenic feature of HSP70 comes from its ability to bind antigenic peptides derived from tumors.^337^ However, HSP70 or the 14‐mer HSP70 peptide (TKDNNLLGRFELSG, TKD) alone is not sufficient to stimulate NK cells, and the presence of IL‐2 or IL‐15 is also required.^338^ Importantly, the types and dosage of HSP70s can also affect immune responses. The extracellular HSP70 (eHSP70) activates T regulatory cells (Treg) and interacts with APC, suggesting that HSP70s trigger both innate and adaptive immune responses.^339^ In addition, HSP70‐positive exosomes were shown to activate myeloid‐derived suppressor cells (MDSCs), leading to IL‐6 production.^340^ These experiments opened new perspectives for the utilizing of HSP70 as an adjuvant for tumor immunotherapy. HSP70 functions in different states through its catalytic cycle, suggesting that it can be multifunctional in malignant cells. In cancer patients, tumor cells release large amounts of HSP70 into the extracellular microenvironment, which can result in different outcomes for patient survival.^341^ Given its clinical significance, many small‐molecule inhibitors targeting HSP70 were developed.^342^, ^343^, ^344^ In addition, several HSP70‐based immunotherapy approaches have been evaluated in clinical trials.^345^

HSP60 or HSPD1, located on the surface of exosomes secreted by tumor cells but not normal cells, was reported as one of the key players during the progress of cancer. Studies demonstrated that exosomal HSP60 has high potential for clinical use, such as a potential clinical biomarker for diagnosing, monitoring disease progression, and assessing prognosis of a variety of cancers.^260^, ^261^, ^262^ Research showed that HSP60 is increased in patient tumor–tissue samples or EXs obtained from the blood of patients, and some groups are evaluating the application of potential HSP60 inhibitors for treatment of certain cancers,^262^ induced a decrease in the HSP60 levels or its PTMs leading to cell death.^263^ These findings suggest HSP60 has potential clinical applications of both diagnosis and therapy for cancers.

HSPgp96, or GRP94, is one of the HSP90 family members that promotes survival signaling of cancer cells through its multiple client proteins, including HER2, integrins, TLRs, LRP6, and IGF.^264^, ^265^ Recently, studies showed that HSP gp96 expression positive (>90%) was an independent poor prognostic predictor in gallbladder cancers.^346^ Gp96 forms a complex with tumor antigens through acting as a molecular chaperone before the APCs incorporate the antigen, which is then incorporated into dendritic cells via gp96 receptor.^347^ Some studies documented that gp96 purified from tumors was able to initiate efficient antitumor responses and protective immunity in both animal models and clinical patients, suggesting that gp96 worked in both preventive and therapeutic protocols.^264^, ^323^, ^348^ Therefore, gp96 plays a critical role in antitumor immunity and is now being studied in vaccine applications to treat cancers.

Other HSPs also participate in cancer development in various ways. HSF1 is a chaperone that associates with both HSP70 and HSP90, and it has been shown to play a significant role in different cancers based on its functions in supporting cell migration, invasion, proliferation, and cancer cell metabolism.^266^ Studies in a broad spectrum of cancers showed that active HSF1 is located in the cell nucleus and is associated with poor prognosis.^267^ Moreover, genetic knockdown studies of HSF1 have validated the targeted effects in cancers, suggesting HSF1 as a potential therapeutic target.^268^ In addition, HSF1 was investigated as a biomarker for patient outcomes in various cancers.^266^

Small chaperone HSP27 acts as an ATP‐independent chaperone with potent antiapoptotic property and is involved in cell growth, differentiation, migration, and tumor progression.^269^ However, the protective or counter‐protective capabilities of HSP27 in different diseases make it a controversial drug target.^270^ Studies showed an aberrant high level of HSP27 in various types of cancers, such as prostate, brain, ovarian, and breast cancers. Recently, research on HSP27 is being conducted to elucidate its possible contributions in invasion and metastasis cascade that affect the overall patient survival, and the results indicated a possible association of HSP27 levels with poor prognosis.^269^, ^270^, ^332^ There are also studies showing that HSP27 levels in serum and in TME were significantly higher in both PC and breast cancer patients, whereas low level of HSP27 was associated with nonresponsiveness to chemotherapy treatment, suggesting that HSP27 could be used as a biomarker for the diagnosis of certain human cancer.^271^, ^272^ In terms of drug development, there are now some preliminary trials targeting HSP27 for cancer therapy, primarily by downregulating HSP27.^349^ However, unlike other HSPs, HSP27 does not bind ATP, making HSP27 difficult to target with small compounds.

HSP40 represents a large and poorly studied family of cochaperones. It was reported that the HSP40 family regulates HSP70 function and usually acts together with HSP90 and HSP70 to participate in different cell survival signal pathways.^273^, ^274^ Current studies showed that HSP40 upregulated in both brain tumor and lung cancer, and the levels of HSP40 in the serum of cancer patients can be used for tumor diagnosis. However, a study also uncovered a controversial role of HSP40 in human cancers that confers potential resistance to cytotoxic management of 5‐fluorouracil (5‐FU) and carboplatin.^275^, ^276^ Furthermore, knockdown or inhibition of HSP27 and HSP40 decreases survival of drugs resistant cells.^275^ Further understanding of the role of HSP40 family members in cancer may lead to the discovery of new targets for possible cancer treatment.

### 6.2 HSPs as targets in neurodegeneration diseases

HSPs have recently become a focus of research in neurodegenerative diseases (such as AD, Parkinson's disease, polyglutamine (polyQ) disease, Amyotrophic lateral sclerosis, Huntington's, and many others) because the pathogenesis of these diseases is highlighted by the intracellular proteins aberrant folding and inclusion body formation.^282^ HSPs serve as protein‐folding machinery and work together with UPS to assist in removing aberrant proteins, exert antiapoptotic effects, and maintain the dynamic homeostasis of dopaminergic neurons against stress conditions such as oxidative damage. Dysfunction of HSPs, including HSP90, HSP70, and HSP27, may contribute to the pathogenesis of Parkinson's disease as a study showed, suggesting that HSPs may come to be potential therapeutic targets for neurodegenerative disorders such as PD.^350^

Overexpression of HSP90 has been reported to reduce the inclusion and accumulation of pathogenic proteins, and to improve phenotypes in neuronal cells and mouse models.^351^ Studies have shown that HSP90/P23/Pin1 complex promotes the dephosphorylation and refolding of tau.^277^ However, when the refolding process is not complete, tau is transferred to the HSP70/CHIP complex and further ubiquitination system mediates their degradation.^278^ HSP90 inhibitors also exert treatment effects through selective mediate proteasomal degradation of their client proteins. In neurodegenerative diseases, HSP90 inhibitors significantly not only reduce the total amount of phosphorylated tau through inducing p35 degradation, but also reduce aggregated forms of tau.^277^, ^279^ Mutant leucine‐rich repeat kinase 2 (LRRK2) is another client protein of Hsp90, and HSP90 inhibitors rescued the axon growth retardation caused by the overexpression of G2019S mutant LRRK2 in neurons.^280^ These studies indicated HSP90 as a therapeutic target of neurodegeneration diseases. Elucidation of its pathophysiological functions using animal models has led to the development of Hsp90 inhibitors and HSP inducers (e.g., Arimoclomol), which suppress the pathogenic process of neuronal degeneration and show clinical effects.^352^

The elevation of HSP70 levels has a neuroprotective effect in some animal neurodegeneration models as indicated in several studies.^353^, ^354^ Recently, studies showed that during the exercise, the released extracellular HSPs 70 (eHSP70) can be internalized by the motoneurons, and then act as intracellular chaperons, protecting cells against protein denaturation, whereas reduced expression of inducible HSP 70 (iHSP70) is associated with neurodegenerative diseases, stressing the importance of understanding the physiological function of extracellular HSP70 in the treatment of neurodegenerative and other neuronal diseases.^355^ Moreover, many studies shown that HSP70 may solubilize α‐syn and promote the degradation of its insoluble forms via chaperone‐mediated autophagy and the proteasome pathway in PD.^281^, ^282^ In addition, other studies suggested that HSP70 plays a role in neuroprotective in diseases that affect CNS and showed promising outcome in reducing the loss of neuron cells.^356^ Therefore, future strategies for developing treatments for neurodegenerative diseases through targeting HSP70 can be proposed. Researchers have already showed that the pharmacological HSP70 activators exhibited beneficial effects in neuronal cells during neurodegenerative‐inducing diseases models by intranasal administration of full‐length recombinant human HSP70 protein, which improved the survival of injured neurons by inducing the production of endogenous HSP70.^357^, ^358^ However, several studies also showed that in the brains of AD patients, neuroprotective functions of molecules such as HSP70 partners are modest. It is clear that the expression and functionality of the inducible forms of HSP70, as well as other important HSPs, are reduced in aged tissues, which needs to be sorted out to ensure that these agents provide therapeutic benefit in neurodegeneration diseases in the future.^283^

The expression level of HSP27 in neurons is low but can be increased by proteotoxic stress. In neurodegenerative diseases, elevated levels of HSP27 in glia and neurons correlate with pathogenic deposition of aberrant proteins and showed protective effects in neuronal cells.^283^ HSP27 acts as a mediator in the survival response to central nervous system (CNS) injury with its antioxidant activity and ability to inhibit cell death pathways. One study demonstrated the role of HSP27 in AD through modulating tau dynamics and assisting in tau clearance from the brain.^284^ Other studies uncovered the role of HSP27 in regulating Amyloid β (Aβ) peptides that is associated with senile plaques in AD brain tissue in in vitro study, and in reducing α‐syn aggregation in Lewy Bodies.^285^, ^286^ Moreover, several studies in animal models revealed the potential involvement of HSP27 in peripheral nerve injuries, which may yield benefit to selective neuronal loss in AD and PD. For example, one study reported that HSP27 increased axonal growth after peripheral nerve damage and initiated motor regenerative response.^287^ These studies suggest that HSP27 could be a promising new therapeutic target for some neurodegenerative diseases. Therefore, it is feasible to develop agents that can stimulate HSP27 function as a potential therapeutic approach, providing insights for innovative treatments for neurological disorders.

### 6.3 HSPs as targets in cardiovascular diseases

The roles of HSPs in heart function have been extensively studied and recent studies highlighted their therapeutic effects in CVDs. There are numerous studies indicating that HSPs (e.g., HSP22, HSP27, HSP60, and HSP70) play critical roles in protecting cardiomyocytes against stress situations such as hypoxia, ischemia, and infection, suggesting their therapeutic potential for CVDs.^359^, ^360^, ^361^

HSP22 adopts complex mechanisms in cardiomyocyte protection, in that it may activate a number of cell survival pathways, resist reversible damages, and have an antiapoptotic effect. HSP22 expression and phosphorylation increased during the compensatory period of heart failure after myocardial infarction and protect mitochondrial function. At the same time, by regulating proteostasis, HSP22 maintains mitochondrial function and integrity.^288^ In vitro studies revealed that USP22 expression elevated when cardiomyocytes were exposed to hypoxia, thus promoting the expression of NOS in the cytoplasm and mitochondria. More studies showed that HSP22 protects mitochondrial function, inhibits mitochondrial apoptosis pathway, resists oxidative stress, and promotes myocardial cell survival and energy metabolism in animal myocardial infarction models, thereby improving myocardial ischemia and heart failure symptoms after myocardial infarction.^359^, ^360^ In addition, USP22 inhibits the Rho GTPase pathway, which is activated during the onset and progression of atherosclerosis, thus lowers the production of actin stress fibers and inhibits the lowering of peak calcium transients, which stabilizes cardiomyocyte structure and safeguard the atrium's electrophysiology and contractile function.^289^, ^290^ Furthermore, HSP22 promotes glycogen synthesis and provides the necessary resources for cardiomyocytes under stress.^291^ Under normal conditions without stress, the heart structure and function in HSP22 knockout mice are unaffected.^360^, ^362^, ^363^ Together, these findings imply that HSP22 may be a therapeutic target for reversing myocardial remodeling and may play a protective part in the progression of heart failure.

HSP27, a member of the small molecule HSP family, is found in abundance in a variety of malignancies and normal tissues, particularly cardiac tissue. The physiological functions of HSP27 linked to CVD are protecting cells from free radicals, heat, ischemia, and harmful chemicals.^361^ HSP27 translocation from cytoplasm to myofibrils and hyperphosphorylated HSP27 in rat heart under ischemia were confirmed by immune‐hybridization analysis, suggesting a role of HSP27 in myocardial ischemia prevention. Studies showed that patients with atherosclerotic plaques have reduced or no HSP27 production compared to healthy people, according to studies. In patients, HSP27 expression and plasma levels were found to be considerably higher in the apparently normal vascular zone close to the plaque than in the plaque core region.^292^ Other studies showed the elevated expression of HSP27 in patients with dilated cardiomyopathy and heart failure caused by ischemic cardiomyopathy. HSP27 reduced the extent of myocardial infarction inducing by closure of the left anterior descending coronary artery, therefore enhanced cardiac function in the Langendorff perfusion model of isolated mouse hearts, implying that HSP27 may be involved in cardiomyocyte protection during persistent myocardial ischemia.^293^, ^294^, ^295^, ^296^ Angiotensin II (Ang II) has pro‐inflammatory effects in thrombospondin vascular smooth muscle cells (VSMCs) by activating the nuclear factor B pathway, which is regulated by HSP27 and is dependent on the phosphorylation of p65. Extracellular HSP27 also works as a signaling molecule in macrophages, activating the nuclear factor‐B pathway. In addition, by acetylating low‐density lipoprotein, HSP27 inhibits the release of the proinflammatory cytokine IL‐1 from macrophages and enhances the secretion of IL‐10. These findings imply that HSP27 is involved in arterial protection and prevention of arterial wall inflammation.^297^, ^298^, ^299^ Furthermore, acute ischemia can cause increased expression of myocardial HSP27. Under heat stress, the ability of aging rat hearts to synthesize HSP27 was dramatically reduced, suggesting that HSP27 synthesis may be a key element for the aging heart to adapt to stress.^364^, ^365^ Therefore, HSP27 is considered to be a potential therapeutic target for CVDs.

HSP60 is mostly found in the mitochondria, where it aids in the normal transport, folding, and assembly of cellular polypeptides or proteins. Overexpressed HSP60 can be recognized as a self‐antigen by the immune system upon stress to boost the body's immunological response, or plays a role in immune signal transduction as a signal molecule. Some groups showed that HSP60 expression is strongly correlated with the degree of atherosclerotic lesions and thickness of the intima, suggesting that HSP60 may play a role in the development of atherosclerosis. Other studies showed that during infection and inflammation, the pro‐atherosclerotic effects induce HSP60 expression, thus causing autoimmune responses and leading to vasculitis and atherosclerosis.^300^, ^301^ However, the mechanism behind remains unclear.^302^ Moreover, HSP60 antibody levels in serum have been linked to the occurrence of coronary atherosclerotic heart disease, suggesting a correlation with the prognosis of severe atherosclerosis.^303^ In addition, some studies in animal models revealed that HSP60 may play a role in dendritic cell antigen presentation and the production of costimulatory molecules in secondary atherosclerosis.^304^

Recently, expression of myocardial HSP70 was detected in the myocardial infarction area in the rat heat shock model, thus promoting the recovery of myocardial contractile. This suggests that HSP70 has effects in myocardial protection and acts as a biomarker of stress or injury for cardiac cells.^366^ The highly expressed HSP70 in rat cardiac tissue reduces NO generation by regulating the activation of inducible NOS, which can increase myocardial cell tolerance to ischemia, suggesting that HSP70 has high protective effects in both ischemia and reperfusion stages of myocardial ischemia.^367^ Moreover, a study in 80 patients having cardiac surgery showed that HSP70 expression in cardiomyocytes was considerably elevated in patients without atrial fibrillation, imply that inducing HSP70 expression before surgery could be an effective way to prevent postsurgery atrial fibrillation and provide a novel strategy to myocardial protection.^368^, ^369^ Another group found that HSP70 can increase myocardial contractility and exert myocardial protection through enhancing the internal environment of ischemic myocardium Ca2+ channels and reducing cardiac Ca2+ overload.^305^ There are also studies reporting the HSP70's cardioprotective function in controlling of ATP‐sensitive potassium channels. And HSP70 protects the heart against emergency‐induced harm through transporting between cytoplasm to nucleus, resulting in aberrant electrical activity of the heart in an injured state.^306^, ^307^ In addition, several studies investigated the pathogen load, HSP70 antibody, and serum HSP70 in patients with coronary heart disease, and demonstrated that decrease expression of HSP70 is a risk factor for coronary heart disease, whereas increasing HSP70 levels may help to prevent atherosclerosis, suggesting that HSP70 may be a therapeutic target for atherosclerosis.^370^, ^371^ Similarly, HSP70 antibody and HSP70 expression levels can be utilized as biomarkers for diagnosis of acute coronary syndrome.^372^, ^373^, ^374^

### 6.4 HSPs as targets in autoimmune diseases, inflammation, and infection diseases

HSPs are endogenous adjuvants that induce strong tumor‐specific and pathogen‐specific immunity. Recent studies showed that some HSPs are secreted extracellularly during stress responses, prompting the immune system to respond to adverse cellular conditions. HSPs are associated with both pro‐ and anti‐inflammatory responses, and their effects on immune cells depend on the concentration of the respective HSPs secreted from cancer cells.^375^, ^376^

The correlation between HSPs and autoimmunity is complex although vaccination with HSPs could protect animals from autoimmune diseases. The high homology between human and microbial HSPs could cause autoimmune disorders through immune cross‐reactivity. Studies showed that HSPs could be involved in many autoimmune diseases, including Behcet's disease (BD), type 1 diabetes mellitus (T1DM), arthritis, and systemic lupus erythematosus. For example, human HSP60 is implicated in pro‐inflammation by stimulating macrophages to secrete IL‐6; TNF‐α, IL‐12, and IL‐15 in Type 2 diabetes.^308^ HSP60 was also involved in the development of autoimmune encephalomyelitis in rats, but the mechanism is not clear.^309^ Several studies discovered that antibodies reacting with HSP60 from various infectious agents (e.g., microorganisms) were found in patients suffering from spondylarthrosis, suggesting a role of HSP60 in autoimmune diseases involving infectious agents.^310^, ^311^ Therefore, some studies have reported the intervention strategies targeting HSP60 function in various human inflammatory diseases. For instance, the regulatory activities of HSP60 (the p277 peptide from human HSP60) were used to treat human type 1 diabetes (T1D) patients.^377^

The molecular chaperones that play roles in signal transduction processes and immune regulation in general have also been reported.^378^ For example, several HSPs, such as HSP90, HSP70, HSP60, HSP40, and HSP gp96, were shown to be involved in the production of pro‐inflammatory cytokines.^379^ Studies also showed that some of the cytokines are secreted in response to the presence of HSPs, including IL‐1, IL‐6, IL‐12, TNF‐α, and anti‐inflammatory cytokines such as IL‐10.^379^ Furthermore, some HSPs induce the release of chemokines from immune cells, and even modulate the maturation of DC cells.^378^

Studies also indicate that HSPs facilitate the folding of receptors on immune cells, thus activating the innate immune response and eventually the adaptive immune system. For example, HSP90 serves as a signal to alert tumor‐immune response and is involved in antigen presentation.^312^ The recognition of HSP70 by immune cells initiates signal transduction, resulting in the subsequent release of cytokines.^313^ HSP70 was also shown to promote the production of anti‐inflammatory cytokines in chronic inflammatory diseases.^314^ Moreover, HSP90 inhibition could lead to elevated expression of interferon response genes during tumor immunotherapy.^315^ Together, these studies suggest that some HSPs could be potential therapeutic targets for both human autoimmune diseases and inflammations.

Microbial infections, such as pathogenic bacteria, viruses, parasites, and fungi, could put severe stress to cells, and the role of HSPs in infection is another active research area.^380^ Organisms such as fungi and virus may express molecular chaperons such as HSPs to perform essential biological functions.^381^ HSP90 was indicated as a potential antifungal target according to its role in morphogenesis.^316^ Elevated HSP70 expression was observed in the lymphocytes of human immunodeficiency virus (HIV)‐positive patients.^317^ Microbial HSP70 and the peptide epitope (aa 407–426) was capable of efficiently inhibiting type‐1 HIV infection of human CD4+ T cells.^318^ One study discovered a significant correlation between the level of HSP gp96 and progression of diseases caused by hepatitis B virus.^319^ Moreover, HSP40 was shown to stimulate Th1 and Th17 immune responses against Streptococcus pneumoniae infection in mice.^320^ Therefore, HSPs inhibitors or vaccines were suggested as therapeutic agents for different virus infection diseases (e.g., HSP90 inhibitors for picornavirus infection, HSP70 vaccines for HSV and HIV‐1).^382^, ^383^, ^384^ Together, these studies demonstrated that HSPs could be valuable therapeutic targets for infection diseases.

### 6.5 HSPs as disease biomarkers

In the development of targeted therapies and drugs, it is critical to select patients whose diseases depend on these targets prior to treatment so that they can benefit the most from the treatment. Hence, there is a pressing need to identify relevant biomarkers and develop diagnostic tools capable of identifying appropriate patients prior to clinical trials and giving the greatest chance for successful outcome. As mentioned above, some HSPs expression is higher in disease states. For example, HSP90, HSP70, HSP60, HSP27, and HSF1 have been investigated as potential biomarkers for patient outcomes in multiple cancers, including lung cancer, breast cancer, CRC, pancreatic carcinoma, hepatocellular carcinoma, ovarian cancer, PC, and leukemia.^112^, ^266^, ^332^, ^335^, ^385^ The overexpression and crucial roles of HSPs in various cancers make them tumor markers for targeted drug delivery. Studies have shown that anti‐HSP therapy exhibits significant antitumor activity against human prostate and breast cancers in different clinical trials. For example, as a relevant biomarker for tumor metastasis, the synthetic heat shock protein HSP78 (GRP78)‐binding peptides conjugated with programmed cell death sequence can impair both prostate and breast cancer cell growth in different models.^386^ The identified GRP78‐specific peptide, including WDLAWMFRLPVG and WIFPWIQL, were applied for targeted delivery of cytotoxic agents to tumor cells that overexpressed GRP78, such as PCs.^386^ Moreover, the nanocarrier‐conjugated scFvs, such as quantum dot conjugated GRP78 scFv, exhibit biological antitumor activity in breast cancer models, suggesting the application as a therapeutic antibody for cancer treatment.^387^ In addition, some studies identified the TME‐targeting nanoparticles (NPs), HSP‐NPs, which based on a natural Methanococcus jannaschii small HSPs (Mj‐sHSPs), and showed the ability in improving therapeutic efficacy and decreasing chemotherapy adverse effect in tumor therapy.^388^

Besides the function of HSPs as target for drug delivery in cancers, studies also uncovered roles of targeting of HSPs in many other diseases. For example, targeted mitochondrial HSP90 with an inhibitor Gamitrinib‐triphenylphosphonium (G‐TPP) can chemically interfere mitochondrial protein folding, thus relieving Parkinson's disease by inducing mitophagy.^389^ Some studies showed that the levels of host HSP65 and HSP71 can be used for the diagnosis of active tuberculosis (TB), whereas HSP16 was more specific for diseases in latency and was used as a diagnostic marker for latent tuberculosis infection (LTBI).^390^ In multiple sclerosis (MS), HSP60 was reported as a useful biomarker for progression.^391^ In addition, HSP70 antibody and the plasma concentrations of HSP70 were shown as potential screening biomarkers for early diagnosis of heart failure, including acute coronary syndrome.^372^, ^373^, ^374^, ^392^ Together, these studies demonstrated the important role of HSPs in disease diagnosis as clinical biomarkers.

However, unlike tyrosine kinase inhibitors targeting overexpressed or mutated kinases that could directly confer drug sensitivity in cells, the context in which these HSP drugs could be used is complicated. Previous studies uncovered that it is not only the levels of HSPs in different related diseases (e.g., cancer), but also the functional status as determined by complex formation as well as PTMs that determine sensitivity to inhibitors.^263^, ^330^ Therefore, a comprehensive understanding and discovery of other related clinical biomarkers may significantly improve patient treatment.

## 7 DRUG DISCOVERY FOR HSPSs

Oncogenic HSPs have been recognized as potential therapeutic targets and clinical biomarkers for diagnosis and prognosis for a lot of diseases associated with aberrant protein signaling. Until now, HSP90 and HSP70 have been extensively investigated in terms of drug discovery and they have received the most interest in the past two decades. Up to now, the development of HSP90 inhibitors is the most advanced among HSPs, and more than 20 inhibitors that have undergone clinical trials, some of which are limited by adverse toxicities during several clinical evaluation and none has been approved by the FDA yet. Other HSPs, including HSP70, gp96, and HSP27, are being studied in vaccines or antisense oligonucleotides to treat disease like cancers (Table 5).^323^, ^324^

**TABLE 5:** Current status of HSP inhibitors and application in clinical trials^a^

Drugs	Target	Conditions	Clinical trial phase
Geldanamycin	HSP90	Advanced solid tumors or non‐Hodgkin's lymphoma (NCT00019708, NCT00003969)	Phase I terminated or completed
Tanespimycin	HSP90	Inoperable locoregionally advanced or metastatic thyroid cancer (NCT00118248)	Phase II completed
		With hormone‐resistant prostate cancer (NCT00564928)	Phase II completed
Alvespimycin	HSP90	Relapsed chronic lymphocytic leukemia, small lymphocytic lymphoma, or B‐cell prolymphocytic leukemia (NCT01126502)	Phase I terminated or completed
		Metastatic or unresectable solid tumors or lymphomas (NCT00088868)	Phase I completed
		Her2 positive breast cancer (NCT00803556)	Phase II terminated
Retaspimycin	HSP90	Nonsmall cell lung cancer (NCT01427946), hormone‐resistant prostate cancer (NCT00564928)	Phase I/II completed
		Inoperable locoregionally advanced or metastatic thyroid cancer (NCT00113204)	Phase II completed
IPI‐493	HSP90	Hormone‐resistant prostate cancer (NCT00564928)	Phase II completed
		Hematologic malignancies (NCT01193491)	Phase I terminated
		Combination with everolimus in KRAS mutant non‐small cell lung cancer (NCT01427946)	Phase I/II completed
		Trastuzumab pretreated, locally advanced or metastatic HER2 positive breast cancer (NCT00817362)	Phase II terminated
Luminespib (NVP‐AUY922)	HSP90	Advanced solid malignancies (NCT01602627)	Phase I terminated
		Metastatic pancreatic cancer who are resistant to first line chemotherapy (NCT01484860)	Phase II terminated
		GIST (gastrointestinal stromal tumor) patients (NCT01389583)	Unknown
Onalespib AT13387	HSP90	Prostate cancer (NCT01685268)	Phase I/II completed
		Relapsed/refractory ALK+ anaplastic large cell lymphoma (ALCL), mantle cell lymphoma (MCL), and BCL6+ diffuse large B cell lymphoma (DLBCL) (NCT02572453)	Phase II terminated
		BRAF V600E mutation present (NCT02097225), advanced triple negative breast cancer (NCT02474173)	Phase I active, not recruiting
Ganetespib (STA‐9090)	HSP90	Advanced hepatocellular cancer (NCT01665937), metastatic breast cancer (NCT01273896), advanced esophagogastric cancer (NCT01167114), metastatic ocular melanoma (NCT01200238), relapsed or refractory small cell lung cancer (NCT01173523)	Phase II completed
		Combined with Crizotinib in ALK positive lung cancers (NCT01579994)	Phase I Completed
Zelavespib (PU‐H71)	HSP90	Metastatic breast cancer (NCT03166085), Myelofibrosis (NCT03373877)	Phase I terminated
		Advanced malignancies (NCT01393509)	Phase I active, not recruiting
Icapamespib (PU‐AD, PU‐HZ151)	HSP90	Amyotrophic lateral sclerosis (ALS) (NCT04505358)	Phase II withdrawn
		Recurrent malignant glioma (Glio) (NCT04782609)	Phase I recruiting
MPC‐3100	HSP90	Safety study in cancer patients who have failed other treatments (NCT00920205)	Phase I completed
Debio0932 (CUDC‐305)	HSP90	Nonsmall cell lung cancer, advanced solid tumors or lymphoma (NCT01168752)	Phase I completed
BIIB021 CNF2024	HSP90	Advanced solid tumors (NCT01017198)	Phase I completed
BIIB028	HSP90	Solid tumors (NCT00725933)	Phase I completed
SNX‐2112	HSP90	TP53 null cancers (NCT02612285)	Phase II terminated
SNX‐5422 PF‐04929113	HSP90	TP53 null cancers (NCT02612285)	Phase II terminated
		Human epidermal growth factor receptor 2 (HER2) positive cancers (NCT01848756)	Phase I/II terminated
		Solid tumor cancers and lymphomas, refractory hematological malignancies (NCT01635712), refractory solid tumor malignancies (NCT01892046)	Phase I Completed
VER‐82576 NVP‐BEP800	HSP90	Acute lymphoblastique leukemia (NCT04437420)	Recruiting
Autologous HSP70‐peptide complex in combination with imatinib mesylate	HSP70	Chronic myeloid leukemia (NCT00058747)	Phase II
Autologous HSP70‐peptide complex (AG‐858) in combination with Gleevec	HSP70	Chronic myeloid leukemia (NCT00058747)	Phase II

^a^ The data were obtained from https://www.clinicaltrials.gov/.

### 7.1 HSP90 small molecule inhibitors

#### 7.1.1 Geldanamycin analogues

GM is a benzoquinone‐derivative ansamycin antibiotic. GM was first reported in 1970 from a Streptomyces species, and inhibits the ATPase activity of Hsp90 with a nanomolar IC_50_, the binding mode was in the ATP‐pocket of HSP90 by mimicking the nucleotide. However, severe hepatotoxicity and poor solubility affected the conduct of clinical trials. 17‐AAG (tanespimycin) and 17‐DMAG (alvespimycin) are both the GM derivatives, which were obtained by replacement of the 17‐methoxy group with the different amine groups. In 1999, tanespimycin entered clinic as the first HSP90 inhibitor, and showed good therapeutic effect in various cancers either in single dose or in combinations, including melanoma,^393^ ERBB2‐positive metastatic breast cancer,^394^ refractory MM,^395^ and so on. Unfortunately, due to the same shortcomings of GM such as the hepatotoxicity and poor solubility, the clinical trials of Alvespimycin had to be terminated.^396^ By replacing the methoxy group with N, N‐dimethylpropan‐1‐amino moiety, the solubility, and oral bioavailability of Alvespimycin were improved. Subsequently, Alvespimycin was used in castration‐resistant PC, melanoma, renal cancer, chondrosarcoma,^396^ and breast cancer.^397^ The reduction of quinone to hydroquinone by reductase NQO1 (NADPH/quinone oxidoreductase I) causes the metabolic instability and hepatotoxicity.^398^ By replacing quinone with hydroquinone, Retaspimycin was developed and the solubility can be improved in hydrochloride form,^399^, ^400^ and it was approved to enter clinic. In addition, IPI‐493 was a dealkylation compound of tanespimycin and was now being used in clinical practice (Figure S1A).^401^

#### 7.1.2 Resorcinol‐based HSP90 inhibitors

To overcome the limitations of GM analogs, such as hepatotoxicity and solubility, the resorcinol‐based HSP90 inhibitors were developed (Figure S1B). The resorcinol moiety of radicicol binds deeply of the ATP‐binding pocket of HSP90, and at the same time anchored by H_2_O‐mediated hydrogen bonds. Luminespib (NVP‐AUY922) was developed based on a previously discovered a resorcinol‐structural compound using structure‐based design approach,^402^ and it showed high affinity to Hsp90. Luminespib can induce the proteasomal degradation of oncogenic client proteins^403^ and was evaluated in multiple clinical trials. AT13387 was also a resorcinol‐based HSP90 inhibitor, which was optimized based on a potent lead compound using a fragment‐based design approach,^404^ and it exhibited high affinity of HSP90 through its binding in the ATPase site of the N‐domain. AT13387 inhibited the growth, migration, and clone formation, and eventually showed significant antitumor effect in numerous in vitro and in vivo cancer models.^405^, ^406^ Recently, it was discovered that AT1387 can protect the Alveolo–Capillary Barrier and prevent HCl‐induced chronic lung injury and pulmonary fibrosis at nontoxic low doses.^407^ Ganetespib (formerly known as STA‐9090) is a potent and selective Hsp90 inhibitor containing a resorcinol ring and a unique Triazolone,^408^ and it is currently being evaluated in multiple clinical trials.^408^, ^409^

#### 7.1.3 Purine‐based HSP90 inhibitors

Many HSP90 inhibitors were developed based on the purine ring of natural ligand ATP (Figure S1C). Substitution at C‐8 of the purine moiety was identified as a critical site for optimization, and a phenyl sulfide and amine in the alkyl chain were introduced at C‐8 and N‐9 of the purine ring. Zelavespib (PU‐H71) is also a purine‐based Hsp90 inhibitor,^410^ which can specifically inhibit Hsp90 and chaperone’ function, promote the degradation of oncogenic signaling proteins, and eventually prevent tumor cell proliferation and survival. In 2011, Zelavespib (PU‐H71) was used for the treatment of lymphoma, solid tumors, metastatic solid tumor, myeloproliferative neoplasms (MPNs) in clinical trials, and recently was used in PML‐SYK fusion AML.^411^ However, Zelavespib is unable to permeate the blood–brain barrier (BBB), which rendered it unfeasible for the treatment of diseases in CNS. Icapamespib (PU‐AD, PU‐HZ151) is developed based on the structure of Zelavespib (PU‐H71) aiming to overcome the limitation of BBB with the alkyl chain of the N9‐chain amine moiety modified from isopropyl to dimethylpropyl.^412^ Zelavespib was used for the treatment of Alzheimer's disease (NCT03935568), PET imaging (NCT03371420), and recurrent GBM (NCT04782609). Based on Zelavespib, MPC‐3100 was later developed as a new clinical candidate with the N‐9 alkyl and the iodo of C‐8 arylsulfide modified to N‐substituted piperidine moiety and bromo, respectively. The potency and pharmacokinetic properties of MPC‐3100 were both improved,^413^ and was used for the treatment of cancers (NCT00920205). Debio 0932 (CUDC‐305) was an Hsp90 inhibitor belonging to the purine analogs, the iodo group was changed to N, N‐dimethyl, and the N atom of the purine was removed. Debio 0932 (CUDC‐305) can overcome erlotinib resistance and was used for patients with nonsmall cell lung cancer (NCT01714037) and advanced solid tumors or lymphoma (NCT01168752).^414^ In addition, BIIB021 was developed by replacing aryl substituent at N‐9 with a pyridyl motif and adding a chloro at C‐2 position.^415^ BIIB021 entered clinical trials and showed partial responses in a Phase II trial of gastrointestinal stromal tumors (GISTs).^416^ BIIB028 was further modified based on BIIB021 for improvement in potency, efficacy, and tolerability. In BIIB028, N7 was replaced with a carbon atom, the deeazapurine ring was designed, and a hydroxy alkynes group was substituted at this carbon atom. By adopting a prodrug form using phosphorylation, the solubility of BIIB028 was improved and it can be completely converted into its active metabolite in a short time.^417^ BIIB028 was used as an orally available HSP90 inhibitor for the treatment of advanced solid tumors (NCT00725933).

#### 7.1.4 Other structures HSP90 inhibitors

Although the natural product–derived GM analogs HSP90 inhibitors are efficacious, the hepatotoxicity and lack of oral availability limited the dosing frequency. To overcome this, a novel class Hsp90 inhibitor SNX‐2112 was developed (Figure S1D). SNX‐2112 is an ATP‐competitive Hsp90 inhibitor. SNX‐5422 is the prodrug of SNX‐2112 and highly orally bioavailable with potency against various cancers. Three phase 1 clinical studies of SNX‐5422 are currently recruiting including refractory hematologic and solid tumor malignancies.^418^ NVP‐BEP800 is a novel orally bioavailable ATP‐competitive Hsp90 inhibitor. NVP‐BEP800 was developed through combination of hit identification strategies and the structure‐based medicinal chemistry program, and a new class of 2‐aminothieno[2,3‐d] pyrimidine Hsp90 inhibitors with oral efficacy in animal cancer models was identified.^419^ NVP‐BEP800 showed favorable activity against numerous human tumor cell lines and primary human xenograft models at low concentrations. In clinical evaluation, NVP‐BEP800 was used on two different types of ALL (T‐ and B‐ALL) (NCT04437420). Recently, a novel HSP90 inhibitor (RGRN‐305) was reported for the treatment of plaque psoriasis,^420^ but the structure was undisclosed.

### 7.2 Recombinant peptides or vaccines

#### 7.2.1 Hsp70‐peptide

HSP70 was reported as a stress‐inducible protein with elevated expression in tumor cells but not normal cells. One of well‐developed recombinant peptide in clinical trial is the C‐terminal substrate‐binding domain of HSP70, comprising amino acids 450–461 (aa450–461), termed 14‐mer HSP70 peptide (TKD). TKD stimulated NK cells ex vivo with interleukin‐2 (IL‐2) and showed potential antitumor activity post reinfusion in different animal models or clinical patients.^421^, ^422^ TKD‐treated NK cells bind to tumor cells through structurally recognizing HSP70, thus mediating tumor cell lysis. Moreover, TKD alone is not sufficient to stimulate NK cells, and it also requires the presence of IL‐2 or IL‐15.^338^

Another reported recombinant peptide is chemically identical to or similar to the endogenous HSP70. The autologous vaccination with tumor‐derived HSP70 stimulates the host mice immune system, permits antigen presenting cells (APCs) to present tumor antigens to their cell surface, and directly elicits a tumoricidal cytotoxic T lymphocyte (CTL) response, thereby increasing longevity and survival of tumor‐bearing hosts.^423^

#### 7.2.2 Vitespen, HSP gp96‐peptide complex

Vitespen is an autologous cancer vaccine derived from tumor‐specific HSPgp96 and is currently in different clinical trials (phase II and III) for cancers, including melanoma, CRC, lung cancer, glioblastoma, and renal cell carcinoma. HSPgp96 is a key regulator involved in dendritic cell maturation, migration, antigen processing, as well as T cell activation. Therefore, Vitespen acts as an inducer of major histocompatibility class I‐restricted immune responses in a range of tumor types in preclinical models and showed significant clinical responses in patients with early‐stage disease with few side effects.^424^, ^425^

#### 7.2.3 Apatorsen, HSP27 antisense oligonucleotide OGX‐427

HSP27 has been found to be overexpressed in a variety of human cancers. Apatorsen is a second‐generation antisense oligonucleotide targeting HSP27 mRNA with potential antitumor and chemosensitizing activities. One recent study reported that Apatorsen induced tumor cell apoptosis and enhanced cell cytotoxicity through suppressing HSP27 expression in tumor cells. This potential drug is now in clinical trials for different cancers either alone or in combination treatment, so far it has shown improved outcomes in patients with metastatic NSCLC cancer and platinum‐resistant metastatic urothelial carcinoma.^426^, ^427^ Another study reported the evaluation of Apatorsen in phase II clinical in combination with prednisone for the treatment of castration‐resistant PCs (NCT01120470).

## 8 OUTLOOK

Since the discovery of the heat shock puff in 1962, deeper understanding is achieved about the HSPs structures, functions, and how they cooperate with each other, working as chaperones to orchestrate proteostasis and deal with stresses. However, at the same time, there are still unknown questions about the structures and mechanisms of HSPs. First, the structure mechanisms of how the HSPs work as a network in protein folding is not completely understood, mainly due to the transient and dynamic binding structures of HSPs complexed with clients which are difficult to resolve. Recently, David A. Agard group has resolved the high resolution cryoelectron microscopy structures of Hsp90–p23–GR and Hsp90–Hsp70–Hop–GR, which reveals that the basic mechanism of how the client is loaded to HSP90 from HSP70 and how the cochaperons work in protein folding cycle.^96^, ^97^ Meanwhile, the folding mechanism of large multidomain proteins remains unclear and need better understanding.^428^ Second, the number of proteins in the proteome that rely on HSPs for structure stability still needs to be studied. Although some works has clarified the client proteins of HSP90 in proteomic range,^57^ systematic proteomic studies for the client proteins of other HSPs are still needed. Recently, some principles for HSPs in the recognition of clients are summarized, which may be useful for discover new potential client proteins of HSPs.^37^, ^38^, ^57^, ^429^ As the protein homeostasis is important for cell integrity, survival and metabolism, impairment of chaperone‐assisted protein quality control leads to the onset and development of various diseases.

At the same time, although a lot of studies have proved that HSPs play an important role in the development of diseases, the current drug development for HSPs is not fulfilled. Many HSP90 inhibitors in clinical trials were terminated or delayed due to toxicity or lack of efficacy.^430^ It has observed that the use of HSP90 inhibitors could lead to the activation of HSF1, which, in turn, induce the activation of other HSPs to overcome the lack of HSP90. Therefore, development of inhibitors against multiple HSPs or HSF1 may be a strategy to enhance the efficacy of HSP90 inhibitors and overcome the HSF1‐mediated feedback. In the meantime, we also need to pay attention to the safety of these inhibitors in use. Better understanding of how HSPs function in vivo and the collaboration between the HSPs in cancers will be crucial for reducing toxicity to normal cells and a more accurate indication selection.

## CONFLICT OF INTERESTS

The authors declare no competing interests.

## ETHICS APPROVAL

Not applicable.

## CONTRIBUTIONS

C. Hu, J. Yang, Z.P. Qi, H. Wu, F.M. Zou, B.L. Wang, and H.S. Mei conceived the study, collected the literatures, and drafted the manuscript. Corresponding authors, including W.C. Wang, and Q.S. Liu, provided their corrective comments and tips. J.L revised the manuscript. All authors collaborated to write the article. All authors approved this manuscript for publication.

## Supporting information

Supporting Information

## Data Availability

The data included in this study are available upon request from the corresponding authors.
