# Supplementary material for: Heat shock proteins: Biological functions, pathological roles, and therapeutic opportunities
Source: MedComm (2020). 2022 Aug 2;3(3):e161. doi: 10.1002/mco2.161 (PMC9345296; doi:10.1002/mco2.161)
Supplement: Supplementary file 1 — Supporting Information [file MCO2-3-e161-s001.docx]

**Heat shock proteins: biological functions, pathological roles and therapeutic opportunities**

Chen Hu^1,2^, Jing Yang^1,2^, Ziping Qi^1,2^, Hong Wu^1,2^, Beilei wang^1,2^, Fengming Zou^1,2^, Husheng Mei^1,3^, Jing Liu^1,2,3^, Wenchao Wang^1,2,3*^, Qingsong Liu^1,2,3,4*^

1.Anhui Province Key Laboratory of Medical Physics and Technology, Institute of Health and Medical Technology, Hefei Institutes of Physical Science, Chinese Academy of Sciences, Hefei, Anhui 230031, P. R China

2. Hefei Cancer Hospital, Chinese Academy of Sciences, Hefei, Anhui 230031, P. R. China

3. University of Science and Technology of China, Hefei, Anhui 230026, P. R. China

4. Precision Medicine Research Laboratory of Anhui Province, Hefei, Anhui 230088, P. R. China

*Correspondence: Wenchao Wang (wwcbox@hmfl.ac.cn), Qingsong Liu (qsliu97@hmfl.ac.cn)

**Supplemental Figure 1** Chemical structures of HSP90 inhibitors. A. Geldanamycin analogues; B. Resorcinol-based HSP90 inhibitors; C. Purine-based HSP90 inhibitors; D. Other structures HSP90 inhibitors.
